# Bioinformatic Indications That COPI- and Clathrin-Based Transport Systems Are Not Present in Chloroplasts: An Arabidopsis Model

**DOI:** 10.1371/journal.pone.0104423

**Published:** 2014-08-19

**Authors:** Emelie Lindquist, Mohamed Alezzawi, Henrik Aronsson

**Affiliations:** Department of Biological and Environmental Sciences, University of Gothenburg, Gothenburg, Sweden; University of California - Davis, United States of America

## Abstract

Coated vesicle transport occurs in the cytosol of yeast, mammals and plants. It consists of three different transport systems, the COPI, COPII and clathrin coated vesicles (CCV), all of which participate in the transfer of proteins and lipids between different cytosolic compartments. There are also indications that chloroplasts have a vesicle transport system. Several putative chloroplast-localized proteins, including CPSAR1 and CPRabA5e with similarities to cytosolic COPII transport-related proteins, were detected in previous experimental and bioinformatics studies. These indications raised the hypothesis that a COPI- and/or CCV-related system may be present in chloroplasts, in addition to a COPII-related system. To test this hypothesis we bioinformatically searched for chloroplast proteins that may have similar functions to known cytosolic COPI and CCV components in the model plants *Arabidopsis thaliana* and *Oryza sativa* (subsp. *japonica*) (rice). We found 29 such proteins, based on domain similarity, in Arabidopsis, and 14 in rice. However, many components could not be identified and among the identified most have assigned roles that are not related to either COPI or CCV transport. We conclude that COPII is probably the only active vesicle system in chloroplasts, at least in the model plants. The evolutionary implications of the findings are discussed.

## Introduction

Chloroplasts, the most fully characterised plastids, contain photosynthetically active thylakoids located in an aqueous stroma, surrounded by a double membrane. In addition to the stroma they have two other aqueous compartments: the intermembrane space between the double membrane's outer and inner envelopes, and the lumen enclosed by the thylakoids. Some chloroplast-localized proteins are encoded by the chloroplast genome. However, most (ca. 95%) are encoded by the nuclear genome, processed in the cytoplasm then transferred to chloroplasts [1]. These proteins are translocated across the outer and inner envelope membranes to the stroma via two translocons, designated TOC and TIC, respectively, mostly aided by cleavable transit peptides [2]. However, some non-canonical proteins may enter the chloroplast without a transit peptide. After entering the chloroplast, proteins are further targeted to specific sub-compartments. Thylakoid targeted proteins are transferred from the stroma via one of four pathways: the Secretory (Sec) pathway, the Signal Recognition Particle (SRP) pathway, the Twin Arginine Translocation (Tat) pathway, or the spontaneous pathway. Proteins transported across the thylakoid membrane into the lumen are using the Sec or the Tat pathway, whereas integral thylakoid membrane proteins are using the SRP or the spontaneous pathway [3], [4]. All of these pathways are energy-dependent and mediated by specific combinations of proteins except the spontaneous pathway, which requires no energy inputs or specific proteins for protein transport [5].

Although thylakoid membranes contain proteins their main components are lipids, transferred to the thylakoids after synthesis in the envelope [6], [7]. Several studies indicate that the lipids could be transported by vesicles [8]–[10], but as yet there is no clear evidence of protein transport via vesicles in chloroplasts. In contrast, three coated vesicle transport systems have been characterized in the plant cytosol: the COPII (coat protein complex II), COPI (coat protein complex I) and CCV (clathrin coated vesicle) systems, all similar to corresponding systems in yeast and mammals [11]–[13]. Cytosolic vesicles are known to deliver both soluble and membrane-bound proteins to target membranes, leading to the hypothesis that the vesicle system in chloroplasts may deliver not only lipids, but also proteins [14]. If so, it would represent an uncharacterized fifth pathway for thylakoid-targeted proteins, in addition to the four already identified.

Vesicle transport in chloroplasts has been observed mainly at low temperatures in *Pisum sativum* (pea), *Glycine max* (soybean), *Spinacia oleracea* (spinach) and *Nicotiana tabacum* (tobacco) [15], [16]. Proteins required for vesicle transport in the chloroplast are so far suggested to be similar to those of the well-characterized COPII vesicle transport system in the cytosol [14].

COPII, COPI vesicles and CCV in the cytosol have similar functions, but distinct protein and lipid compositions, and recognize different sets of cargo, which make each transport specific [17], [18]. COPII-coated vesicles appears to be involved exclusively in transport from ER to Golgi [19], [20]. The COPII coat comprises five subunits: Sec23/24, Sec13/31 and Sar1 [11], [21]. Formation of a vesicle starts with activation and recruitment of the small GTPase Sar1 to the donor membrane with the help of Sec12p acting as a guanine nucleotide exchange factor (GEF) at ribosome-free ER membranes in the cytosol [22], [23]. Subsequently coat proteins are gathered and the vesicle is formed. Most cytosol localized coat subunits of COPII have predicted homologs in chloroplasts [14], [24].

Homologues of two important proteins for vesicle transport in the cytosol, RabA5e and Sar1, respectively named CPRabA5e and CPSAR1 (CP = chloroplast localized), have been identified in the chloroplast [9], [14], [25]. CPSAR1 (which has been detected in the envelopes, stroma and stromal vesicles) is required for thylakoid biogenesis, and is more abundant in the envelopes than the stroma at low temperature (4°C), supporting the hypothesis that it participates in a chloroplast vesicle transport system similar to the cytosolic COPII system [9]. CPRabA5e was subsequently identified in chloroplasts showing an attenuation of vesicles and alteration of thylakoid morphology, under oxidative stress [25].

COPI vesicles primarily mediate transport within the Golgi and between the Golgi and ER [11], [26]. The COPI coat (sometimes called coatomer) consists of two main subcomplexes: a cargo-selective F-COPI subcomplex (with *β*, *∂*, *γ and ζ* subunits), and B-COPI subcomplex (with *α*, *β′* and *ε* subunits) [11], [26]. The active form *of* the GTPase ADP-ribosylation factor 1 (Arf1) is needed to initiate coatomer recruitment to Golgi membranes, similarly to the Sar1 requirement for initiation of COPII coat recruitment. Thus, Arf1 and Sar1 act as triggers for COPI- and COPII-coated vesicle maturation, respectively [27].

CCVs play a key role in membrane and protein transport between the trans-Golgi network, plasma membrane and endosomes [26], [28] through the endocytic and late secretory pathways [29]. Their coats consist of clathrin triskelions, structures composed of three “legs” consisting of three heavy chains (each ∼190 kDa) and three light chains (each ∼25 kDa). They form a basket-like lattice of pentagons and hexagons [30], [31] assembled in coordination with other proteins and Arf1. In contrast to COPII and COPI vesicles, adaptor proteins (APs) — including five AP complexes (designated AP1–5), various monomeric adaptors (GGAs) and cargo-specific adaptors — rather than the coat *per se*, are the cargo selectors in CCV vesicles [11], [26], [29]. They bind to membranes and collect cargo to be transported with the vesicles, sometimes forming networks enabling different kinds of cargo to be transported simultaneously [32], [33]. Here we focus on the AP complexes. AP1 and AP2 are dependent on clathrin for vesicle formation, whereas AP3 and AP4 appear to be clathrin-independent [32]. The fifth adaptor protein complex, AP5, was recently discovered in human (HeLa) cells, where it localizes to the endosome and is believed to act independently of clathrin. In this paper vesicles containing these components are collectively referred to as clathrin coated vesicles (CCVs), regardless of their clathrin dependence/independence.

Land plants are known to possess AP1–4, and recent homology analysis suggests they also have AP5 [33]. AP complexes generally consist of four subunits: two large, one medium, and one small [11]. One of the large subunits is called γ, α, δ, ε or ζ, depending on the associated AP complex. The second large subunit is called β and numbered 1–5 depending on the AP complex. Similarly, the medium and small subunits are named μ1–5 and σ1–5, respectively [33]. However, in plants a single subunit called β1/2 probably functions in both AP1 and AP2 complexes, whereas there are distinct β1 and β2 proteins in mammals [34], [35] and σ5 is predicted to be missing from AP5 in Arabidopsis [33]. Like COPI, all AP complexes need Arf proteins for recruitment to membranes [32], [36].

There are numerous similarities in the three vesicle systems, e.g. the requirement for activation of small GTPases (Sar1 in the COPII system, Arf1 in the COPI and CCV systems) for recruitment of the coat and additional proteins [11]. There are also similarities in structural architecture of the coats and the domains they possess. For example, α and β′ subunits of the B-COPI subcomplex form a triskelion similar to clathrin, generating a curved structure. In terms of domain configuration, the β′ subunit of COPI has high similarities to Sec13/31 of COPII, indicating that COPI has similarities to the coats of both CCVs and COPII vesicles [37]. Further similarities include the presence of N-terminal β propellers (enabling binding to AP complexes) and α solenoid legs in the heavy chains of clathrin triskelions of CCVs [37]–[39], Sec13/31 of the COPII coat [40], [41] and the B-COPI subcomplex [26], [37]. In addition, γ and β subunits of the F-COPI subcomplex [26] have similarities to “appendages” of the AP complexes of CCVs, and thus are considered to be cargo-binding [11], [37].

The similarities in, and differences between, the vesicle systems pose intriguing questions about their origin and evolution. COPII is hypothetically the most ancestral system, since it is an essential biosynthetic pathway in all investigated organisms [26], while COPI (which has strong similarities with both COPII and CCV in domain organization and coat structure, respectively) is putatively an intermediate system [37]. If so, the clathrin system evolved most recently.

Current knowledge of chloroplast vesicles indicates that they are most strongly related to the putatively ancestral COPII system [9], [14]. However, the possibility that homologues to cytosolic COPI and CCV systems may be present in chloroplasts has not been systematically explored previously. Thus, we addressed this possibility using the model plant *Arabidopsis thaliana* and yet another model plant *Oryza sativa* (subsp. *japonica*) (rice) to support our findings in Arabidopsis.

## Methods

**Figure 1:**
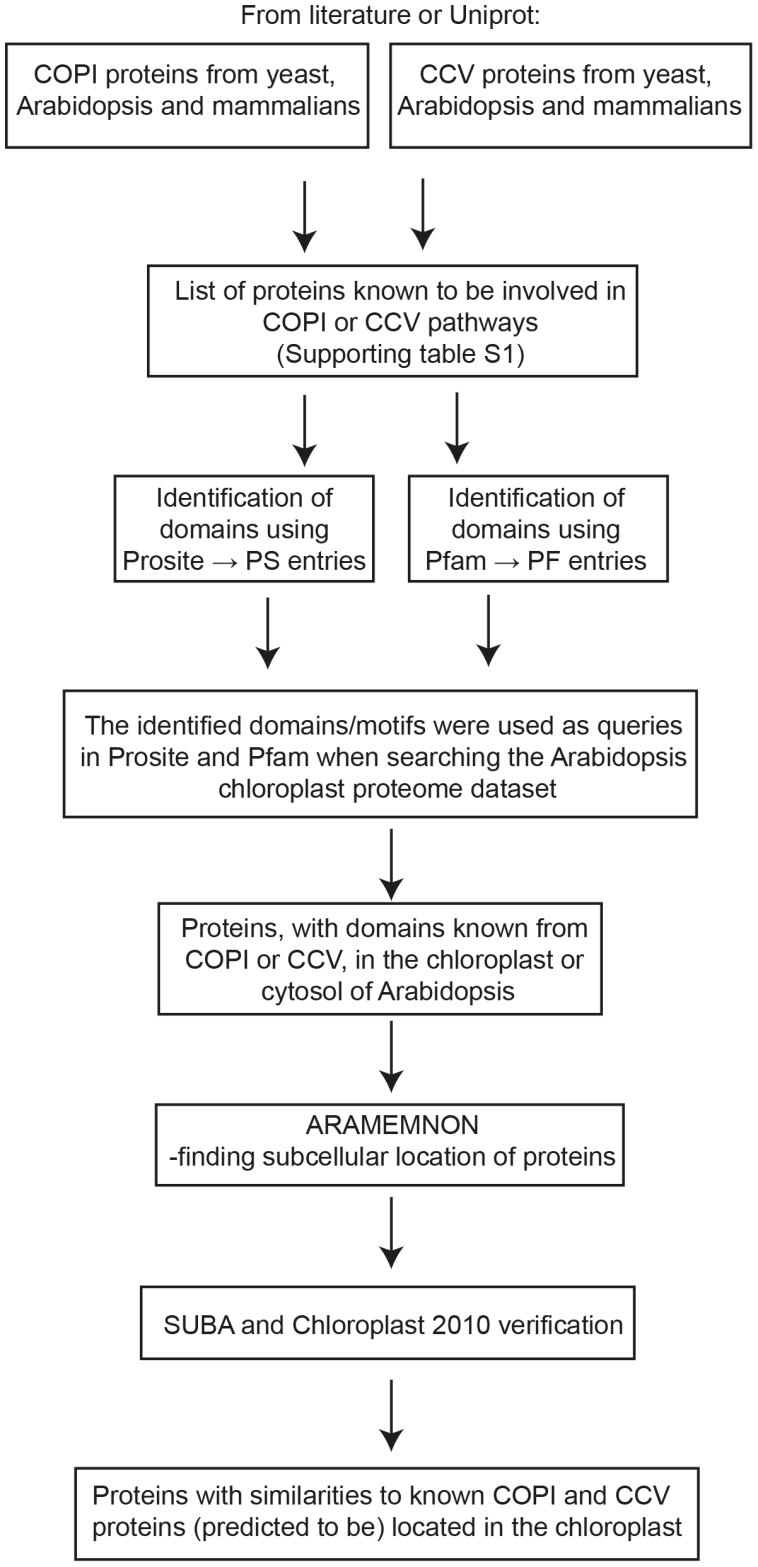
Identification of putative chloroplast COPI and CCV transport components in Arabidopsis. Schematic work flow of the bioinformatics methods used to find putative COPI- and CCV-related transport proteins in chloroplasts. Cytosolic COPI and CCV proteins were retrieved from the literature or Uniprot, and their characteristic domains were identified using Prosite or Pfam. Identified domains were used to search a database of chloroplast-localized proteins, and the localization of proteins found in the chloroplast with relevant domains was further checked using ARAMEMNON, SUBA and Chloroplast 2010. Finally, a list of chloroplast proteins with similarities to known COPI and CCV proteins was compiled.

Multiple *in silico* approaches were used to search for proteins in *Arabidopsis thaliana* chloroplasts that could have homologous functions to COPI and CCV proteins in the cytosol of various organisms. The workflow is presented in Figure 1 and described below.

### Identifying domains, patterns and motifs

Protein sequences matching cytosolic COPI and CCV subunits in *Arabidopsis thaliana*, *Saccharomyces cerevisiae* (Baker's yeast), *Homo sapiens* (human) and *Mus musculus* (mouse) were retrieved from literature and Uniprot (http://www.uniprot.org) (Figure 1, Tables S1, S2, S3, S4, S5, S6, S7, S8, S9). COPII-related proteins were omitted since they were recently investigated [14].

The collected proteins were compiled and searched for characteristic domains, patterns or motifs, using Prosite release 20.95 (http://prosite.expasy.org) [42] and the Pfam database 26.0 (http://pfam.sanger.ac.uk) [43] (Tables S1, S2, S3, S4, S5, S6, S7, S8, S9). Each identified domain, pattern or motif is denoted by either a PS (Prosite) or PF (Pfam) entry.

After converting the dataset of Arabidopsis chloroplast proteins (GO:0009507) (retrieved from TAIR version 10, www.arabidopsis.org) [44] into fasta file format the dataset was searched for the identified Prosite entries using ScanProsite (http://prosite.expasy.org/scanprosite). The Pfam database does not offer corresponding tools so proteins containing the requested Pfam entries were sought manually. The searches generated a list of proteins in Arabidopsis chloroplasts with identical combinations of domains to proteins known to participate in cytosolic COPI or CCV pathways.

As mentioned above, the CCV AP5 complex was first identified in human (HeLa) cells, and subsequently predicted to be present in Arabidopsis, although the degree of conservation is low [33]. Thus, for AP5 we used both human proteins and the predicted proteins in Arabidopsis to identify characteristic domains, which were later used to search the chloroplast dataset.

### Subcellular localization of identified proteins

To further check that identified proteins are localized in chloroplasts we used the ARAMEMNON plant membrane protein database [45] to retrieve their names and predict their subcellular localizations, applying 17 tools provided by the host website (http://aramemnon.uni-koeln.de): BaCelLo [46], ChloroP_v1.1 [47], iPSort [48], Mitopred [49], Mitoprot_v2 [50], MultiLoc [51], PA-SUB_v2.5 [52], PCLR_v0.9 [53], PProwler_v1.1 [54], PrediSi [55], Predotar_v1 [56], PredSL [57], SignalP_HMM_v3 [58], [59], SignalP_NN_v3 [60], SLP-Local [61], TargetP_v1 [60], [62], and WoLF PSort [63]. Using these tools, a Bayesian consensus (SigConsens) score was obtained from ARAMEMNON for each protein with patterns of interest. A score ≥10 was considered reliable, providing a strong prediction of subcellular location [64]. To corroborate the findings we used the SUBA database version 2.21 (http://suba.plantenergy.uwa.edu.au) [65], which in addition to bioinformatics predictions contains information from proteomic and GFP experiments on subcellular localizations of Arabidopsis proteins. Finally, the Chloroplast 2010 database (http://bioinfo.bch.msu.edu/2010_LIMS) [66] was used to confirm the validity of predictions and/or experiments that identified proteins are present in chloroplasts (Figure 1).

### Complementary studies using rice

Complementary investigations of rice (*Oryza sativa* subsp. *japonica*) proteins were conducted to assess the validity and generality of the findings from the Arabidopsis analysis. Domains of relevant Arabidopsis, yeast, mouse and human proteins were retrieved from the Prosite and Pfam websites (Tables S1, S2, S3, S4, S5, S6, S7, S8, S9). Proteins with corresponding combinations of domains were identified in the rice subsp. *japonica* dataset of the National Center for Biotechnology Information (NCBI, TaxID39947) using ScanProsite release 20.102, and their subcellular localizations were predicted using Target P 1.1.

Entries were also searched using Pfam, and proteins with domains of interest were identified manually using a rice dataset downloaded from Phytozome v9.1 (http://www.phytozome.net). To ensure that each hit was from rice subsp. *japonica* they were checked using the Rice genome annotation project (http://rice.plantbiology.msu.edu) or RiceChip Annotation Site (http://www.ricechip.org). Subcellular locations of the hits were then identified using Target P 1.1. Names of identified proteins were retrieved from Uniprot, or the Rice genome annotation project if not identified in Uniprot.

## Results

To find putative components of a hypothetical COPI or CCV system in Arabidopsis chloroplasts, known COPI or CCV proteins from the cytosol of various organisms were retrieved and analysed to identify characteristic domains (Figure 1, Tables S1, S2, S3, S4, S5, S6, S7, S8, S9). The domains were used to search a dataset of protein sequences of chloroplast-localized proteins to identify chloroplast proteins with COPI or CCV domains. Diverse tools, based on differing principles, for predicting the likelihood of proteins having transit peptides were used to strengthen the localization. Several proteins identified in the chloroplast dataset were identical to proteins known to act in the cytosol, raising doubts about their true locations. Occasionally, an identified domain or a domain combination was found in several of the cytosolic proteins, and subsequently in several different chloroplast proteins, hence generating chloroplast proteins which could function as several of the cytosolic subunits. These chloroplast proteins are described below as having commonly occurring domains.

### Putative Clathrin triskelion related chloroplast proteins in Arabidopsis

A protein named Putative heavy chain of clathrin complex (AtCHC2)/At3g08530 was found in the TAIR chloroplast dataset (Table 1) with a Clathrin propeller repeat (PF01394), a Clathrin, heavy-chain linker (PF09268), a Clathrin-H-link (PF13838), a Region in Clathrin and VPS domain (PF00637), and a Clathrin heavy-chain (CHCR) repeat profile (PS50236) as identified in yeast (Table S1). The chloroplast localization of AtCHC2 was also supported by SUBA and Chloroplast 2010 (Table 1).

**Table 1:** Putative chloroplast localized CCV triskelion components identified using characteristic domains in searches of the TAIR chloroplast dataset.

Name (ARAMEMNON), Accession No	Role of chloroplast protein (TAIR)	SigConsens (ARAMEMNON)			SUBA	Chloroplast 2010
		CP	MT	SEC		
**Putative clathrin heavy chain**						
AtCHC2, At3g08530	Protein binding, vesicle transport, endocytosis	0.0	0.0	2.7	Yes (MS/MS)	Yes
**Putative clathrin light chain**						
AtCLC1, At2g40060	Vesicle transport	0.0	0.0	2.0	Yes (MS/MS)	Yes

CP, chloroplast; MT, mitochondria; SEC, secretory pathway.

Similarly, Putative light chain of clathrin complex (AtCLC1/At2g40060; Table 1) was found to have a Clathrin light chain domain (PF01086), identified with known vesicle proteins from both yeast and Arabidopsis (Table S1). Chloroplast localization for this protein was supported by SUBA and Chloroplast 2010 (Table 1).

### Putative Clathrin AP1–5 related chloroplast proteins in Arabidopsis

In clathrin-coated vesicles five AP complexes are known, designated AP1–5. Five proteins similar to the AP1 complex γ subunit were found in the chloroplast dataset: Putative ascorbate peroxidase/At1g07890, Putative thylakoid-bound ascorbate peroxidase (AttAPX)/At1g77490, RNase E/G-type endoribonuclease (AtRNEE/G)/At2g04270, Stromal ascorbate peroxidase (AtsAPX)/At4g08390, and Putative peroxisomal ascorbate peroxidase (AtAPX3)/At4g35000 (Table 2). These five proteins all have the same Peroxidases proximal heme-ligand signature domain (PS00435) as the γ subunit of AP1 in yeast (Table S2). Chloroplast localization was supported for the Putative ascorbate peroxidase and AtAPX3 by Chloroplast 2010, and for AtAPX3 also by SUBA. The other three identified proteins (AttAPX, AtRNEE/G, and AtsAPX) had ARAMEMNON consensus scores >10, indicating a chloroplast location, supported by SUBA and Chloroplast 2010 (Table 2).

**Table 2:** Putative chloroplast localized CCV AP1 complex components identified using characteristic domains in searches of the TAIR chloroplast dataset.

Name (ARAMEMNON), Accession No., subunit	Role of chloroplast protein (TAIR)	SigConsens (ARAMEMNON)			SUBA	Chloroplast 2010
		CP	MT	SEC		
**Putative clathrin AP1 complex protein**						
Putative gamma subunit of coatomer adaptor complex At4g34450*, **β1**	Cytoskeleton organization, protein transport, catabolic processes, vesicle transport	0.4	0.0	4.0	No	No
Unknown protein At1g51350*, **β1**	Unknown	20.4	0.0	3.9	No	Yes
Putative ascorbate peroxidase At1g07890, **γ**	Golgi organization, glycolysis, hyperosmotic response, photorespiration, protein folding (is a ascorbate peroxidase)	0.8	9.0	0.0	No	Yes
AttAPX At1g77490, **γ**	Chloroplast-nucleus signalling, thylakoid membrane organization (is a ascorbate peroxidase)	22.8	5.0	4.9	Yes (MS/MS)	Yes
AtRNEE/G At2g04270, **γ**	Chloroplast mRNA processing, chloroplast organisation, thylakoid membrane organization (is a ribonuclease)	11.2	4.3	0.4	Yes (MS/MS)	Yes
AtsAPX At4g08390, **γ**	Oxidation-reduction processes (is a ascorbate peroxidase)	17.0	5.3	2.2	Yes (MS/MS and GFP)	Yes
AtAPX3 At4g35000, **γ**	Oxidation-reduction processes (is a ascorbate peroxidase)	0.0	5.8	0.0	Yes (MS/MS)	Yes

*contains common occurring domain(s); CP, chloroplast; MT, mitochondria; SEC, secretory pathway.

Considering AP2 homologues, five proteins similar to the β2 subunit in yeast were identified: Putative large subunit of carbamoyl phosphate synthetase VEN3 (AtCarB)/At1g29900, Putative H-protein of glycine decarboxylase/At1g32470, Acetyl-CoA carboxylase (AtACC2)/At1g36180, Biotin carboxylase subunit of plastidic acetyl-coenzyme A carboxylase complex (AtCAC2)/At5g35360, and Putative RimM-like protein involved in 16S rRNA processing/At5g46420 (Table 3). These five proteins all had a Carbamoyl-phosphate synthase subdomain signature 2 (PS00867) identified using Prosite (Table S3).

**Table 3:** Putative chloroplast localized CCV AP2 complex components identified using characteristic domains in searches of the TAIR chloroplast dataset.

Name (ARAMEMNON), Accession No., subunit	Role of chloroplast protein (TAIR)	SigConsens (ARAMEMNON)			SUBA	Chloroplast 2010
		CP	MT	SEC		
**Putative clathrin AP2 complex protein**						
Putative gamma subunit of coatomer adaptor complex At4g34450*, **β2**	Cytoskeleton organization, protein transport, catabolic processes, vesicle transport	0.4	0.0	4.0	No	No
Unknown protein At1g51350*, **β2**	Unknown	20.4	0.0	3.9	No	Yes
Unknown protein At5g57460*, **μ2**	Unknown	6.6	3.0	4.4	Yes (MS/MS)	Yes
AtCarB, At1g29900, **β2**	Response to phosphate starvation, chromatin silencing, gluconeogenesis, metabolic processes	20.1	3.9	0.0	Yes (MS/MS)	Yes
Putative H-protein of glycine decarboxylase, At1g32470, **β2**	Glycine processes, PSII assembly, rRNA processing, biosynthesis of cysteine	8.4	16.2	2.3	Yes (MS/MS)	Yes
AtACC2, At1g36180, **β2**	Fatty acid and metabolic processes (is a acetyl CoA carboxylase)	17.7	6.9	0.2	No	No
AtCAC2, At5g35360, **β2**	Fatty acid and metabolic processes, brassinosteroid and polysaccharide biosynthesis (is a acetyl CoA carboxylase)	20.8	0.0	0.0	Yes (MS/MS)	Yes
Putative RimM-like protein involved in 16S rRNA processing, At5g46420, **β2**	Virus defence, metabolic processes, gene silencing, ribosome biogenesis	14.4	2.7	3.1	Yes (MS/MS)	Yes

*contains common occurring domain(s); CP, chloroplast; MT, mitochondria; SEC, secretory pathway.

Of the five proteins predicted to be chloroplast localized AP2 β2 subunits, three (AtCarB, AtCAC2 and the Putative RimM-like protein involved in 16S rRNA processing) had ARAMEMNON consensus scores >10 and support for this localization from both SUBA and Chloroplast 2010. ARAMEMNON also strongly predicted chloroplast localization for AtACC2, but a mitochondrial location for the Putative H-protein of glycine decarboxylase, although chloroplast localization for the latter was supported by SUBA and Chloroplast 2010 (Table 3).

For AP3, AP4 and AP5 only subunits with commonly occurring domains were identified (Table 4, Tables S4, S5, S6). Further details regarding these proteins are presented below in a separate paragraph.

**Table 4:** Putative chloroplast-localized CCV AP3, AP4 and AP5 complex components identified using characteristic domains in searches of the TAIR chloroplast dataset.

Name (ARAMEMNON), Accession No.	Role of protein (TAIR)	SigConsens (ARAMEMNON)			SUBA	Chloroplast 2010
		CP	MT	SEC		
**Putative clathrin AP3 complex protein**						
**δ and β3 subunit**						
Putative gamma subunit of coatomer adaptor complex, At4g34450*	Cytoskeleton organization, protein transport, catabolic processes, vesicle transport	0.4	0.0	4.0	No	No
Unknown protein, At1g51350	Unknown	20.4	0.0	3.9	No	Yes
**Putative clathrin AP4 complex protein**						
**ε subunit**						
Unknown protein, At5g57460*	Unknown	6.6	3.0	4.4	Yes (MS/MS)	Yes
Putative gamma subunit of coatomer adaptor complex, At4g34450*	Cytoskeleton organization, protein transport, catabolic processes, vesicle transport	0.4	0.0	4.0	No	No
**μ4 and σ4 subunit**						
Unknown protein, At5g57460	Unknown	6.6	3.0	4.4	Yes (MS/MS)	Yes
**Putative clathrin AP5 complex protein**						
**μ5 subunit**						
Unknown protein, At5g57460	Unknown	6.6	3.0	4.4	Yes (MS/MS)	Yes

*contains common occurring domain(s); CP, chloroplast; MT, mitochondria; SEC, secretory pathway.

### Putative B-COPI subcomplex related chloroplast proteins in Arabidopsis

COPI vesicle coats consist of a B-COPI subcomplex and an F-COPI subcomplex, both composed of several subunits (Tables S7, S8). Our searches detected eight proteins similar to the β′ subunit of the B-COPI subcomplex, with a Trp-Asp (WD) repeats circular profile (PS50294) and a Trp-Asp (WD) repeats profile (PS50082), which identifiey the β′ subunit in both Arabidopsis and human cytosol: Receptor for activated C kinase (AtRACK1A)/At1g18080, Putative U-box-type E3 ubiquitin ligase (AtPUB60)/At2g33340, Putative Cdc20-like mitotic specificity factor for anaphase-promoting complex (AtFZR2/AtCCS52A1)/At4g22910, Putative Cdc20-like mitotic specificity factor for anaphase-promoting complex (AtFZR3/AtCCS52B)/At5g13840, WD40 repeat protein, functions in chromatin assembly (AtMSI1)/At5g58230; and three Unknown proteins/At1g24130/At4g02660/At1g15850 (Table 5). Out of these eight proteins only AtFZR2/AtCCS52A1, AtFZR3/AtCCS52B and one of the Unknown proteins (At1g24130) had scores above 10 using ARAMEMNON, and support by Chloroplast 2010. The other five proteins; AtRACK1A, AtPUB60, AtMSI1, and the other two Unknown proteins (At4g02660 and At1g15850), had scores below 10 in ARAMEMNON but were supported as chloroplastic by SUBA and/or Chloroplast 2010 (Table 5).

**Table 5:** Putative chloroplast localized B-COPI components identified using characteristic domains in searches of the TAIR chloroplast dataset.

Name (ARAMEMNON), Accession No.	Role of protein (TAIR)	SigConsens (ARAMEMNON)			SUBA	Chloroplast 2010
		CP	MT	SEC		
**Putative B-COPI subcomplex protein (β′ subunits)**						
AtRACK1A, At1g18080	Response to ABA, GA signalling, glycolysis, translation, salt stress, ribosome biogenesis, seed germination	0.0	0.0	0.0	Yes (MS/MS)	No
AtPUB60, At2g33340	Nucleotide binding	0.0	0.0	0.0	Yes (MS/MS)	Yes
AtFZR2 (AtCCS52A1), At4g22910	Protein binding, cell growth, proteasome assembly, regulation of cell division	11.6	0.0	0.0	No	Yes
AtFZR3 (AtCCS52B), At5g13840	Protein binding, DNA methylation, gamete generation, microtubule organization, proteasome assembly, cell division	18.1	0.0	0.0	No	Yes
Unknown protein, At1g24130	Nucleotide binding	10.8	0.0	0.5	No	Yes
Unknown protein, At4g02660	Signal transduction	0.0	0.0	0.0	Yes (MS/MS)	No
AtMSI1, At5g58230	Protein binding, cell proliferation, chromatin modification, seed development, DNA replication	0.4	0.0	0.0	Yes (MS/MS)	Yes
Unknown protein, At1g15850	Nucleotide binding	9.2	0.0	2.9	No	Yes

CP, chloroplast; MT, mitochondria; SEC, secretory pathway.

### Putative F-COPI subcomplex related chloroplast proteins in Arabidopsis

For the F-COPI subcomplex ζ subunit two proteins were found in the chloroplast: Component of magnesium-protoporphyrin IX chelatase complex (AtCHLD)/At1g08520, and Unknown protein/At1g67120 (Table 6), both having a VWFA domain profile (PS50234) (Table S8). ARAMEMNON strongly predicted chloroplast localization for AtCHLD, but not for the Unknown protein At1g67120, although it was supported for both of these proteins by SUBA and Chloroplast 2010 (Table S8).

**Table 6:** Putative chloroplast localized F-COPI components identified using characteristic domains in searches of the TAIR chloroplast dataset.

Name (ARAMEMNON), Accession No.	Role of protein (TAIR)	SigConsens (ARAMEMNON)			SUBA	Chloroplast 2010
		CP	MT	SEC		
**Putative F-COPI subcomplex protein**						
**ζ subunit**						
AtCHLD, At1g08520*	Chlorophyll biosynthesis, cytokinin metabolic process, photosynthesis	22.8	0.4	0.0	Yes (MS/MS)	Yes
Unknown protein, At1g67120	Cytoskeleton organization, embryo sac development, gluconeogenesis	0.0	1.1	3.2	Yes (MS/MS)	Yes
**γ subunit**						
Putative gamma subunit of coatomer adaptor complex, At4g34450	Cytoskeleton organization, protein transport, catabolic processes, vesicle transport	0.4	0.0	4.0	No	No
**δ subunit**						
Unknown protein, At5g57460	Unknown	6.6	3.0	4.4	Yes (MS/MS)	Yes

*contains common occurring domain(s); CP, chloroplast; MT, mitochondria; SEC, secretory pathway.

### Proteins with commonly occurring domains in Arabidopsis chloroplasts

**Figure 2:**
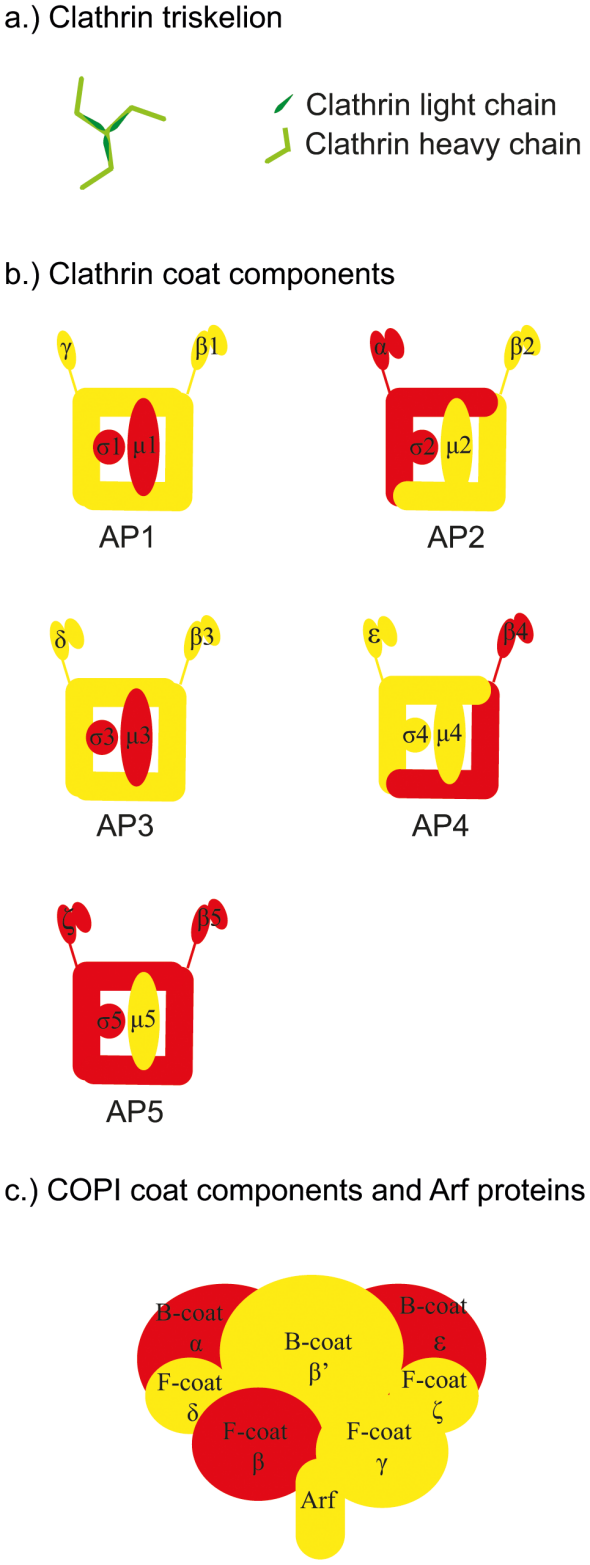
A model of putative CCV and COPI components in Arabidopsis chloroplasts. The figure is based on homologies to components of cytosolic systems (http://www.endocytosis.org/Adaptors/index.html) [130], [131]. Red, no proteins with domains of interest detected in chloroplasts. Yellow, proteins with domains of interest identified in chloroplasts, but known to have other roles than vesicle transport and/or the proteins identified had commonly occurring domains, and thus predicted as different subunits in the chloroplasts, and unknown proteins. Green, proteins with domains of interest found in chloroplasts and previously known to have a vesicle transport role.

Seven proteins in the chloroplast dataset (four of which were potential Coat GTPases) were found to be possible homologues of two or more components of the COPI and CCV system, since some vesicle proteins from the cytosol share the same domain(s) (Tables S2, S3, S4, S5, S6, S8, S9), which thus identify the same proteins in the chloroplast dataset (Figure 2, Tables 2–4, 6–7).

**Table 7:** Putative chloroplast localized CCV and COPI Coat GTPase components identified using characteristic domains in searches of the TAIR chloroplast dataset.

Name (ARAMEMNON), Accession No.	Role of chloroplast protein (TAIR)	SigConsens (ARAMEMNON)			SUBA	Chloroplast 2010
		CP	MT	SEC		
**Putative Coat GTPases**						
AtSARA1A, At1g09180*	Intracellullar transport	0.4	0.0	19.9	Yes (MS/MS)	Yes
AtRabA5e, At1g05810*	Protein transport, GTP mediated signalling	19.2	1.2	2.8	No	Yes
AtRabB1c, At4g35860*	Protein transport, vesicle transport, protein targeting to the vacuole	0.0	0.0	5.9	Yes (MS/MS)	Yes
Putative GTPase of unknown function, At5g57960*	GTP binding	17.3	1.4	0.0	Yes (MS/MS)	Yes

*contains common occurring domain(s); CP, chloroplast; MT, mitochondria; SEC, secretory pathway.

The first is the Putative gamma subunit of coatomer adaptor complex/At4g34450, which has domain homology with the following subunits: AP1 β1, AP2 β2, AP3 δ, AP3 β3, AP4 ε, and F-COPI γ. All these subunits have the same identifying domain, the Adaptin N terminal region (PF01602) (Tables S2, S3, S4, S5, S6, S8), except the F-COPI γ subunit, which also contains the Coatomer gamma subunit appendage platform subdomain (PF08752) (Table S8). Chloroplast localization was very weakly predicted for this protein by ARAMEMNON (consensus score 0.4), and not supported by either SUBA or Chloroplast 2010 (Tables 2–4, 6).

The second protein, an Unknown protein/At1g51350 also contains the PF01602 domain and could function homologously to the AP1 β1, AP2 β2, AP3 δ, AP3 β3 and AP4 ε subunits (Tables 2–4, S2, S3, S4, S5). It has strongly predicted chloroplast localization according to ARAMEMNON, supported by Chloroplast 2010 (Tables 2–4).

The third protein found in the chloroplast dataset that could have several functions was another Unknown protein/At5g57460 with a Mu homology domain (MHD) profile (PS51072), an identifier of AP2 μ2, AP4 μ4, AP4 σ4, AP5 μ5 and F-COPI coat δ subunits (Tables S3, S5, S6, S8). The Unknown protein (At5g57460) is located in chloroplasts according to SUBA and Chloroplast 2010 (Tables 3–4, 6).

### Putative Coat GTPase related chloroplast proteins in Arabidopsis

The last four proteins with commonly occurring domains were identified as putative homologues to Arf proteins. The Arf proteins used as queries in this search were from yeast and the Arabidopsis cytosol, where the latter are divided into four groups (A, B, D and B2) (Bassham et al, 2008). Regardless of their origin, all identified Arf proteins have a small GTPase Arf family profile (PS51417), and an ADP-ribosylation factor family (PF00025) domain (Table S9). Since there was no distinction in the domains identifying the known proteins, the chloroplast search recognized the same four proteins (At1g09180, At1g05810, At4g35860 and At5g57960), regardless of which Arf protein used as a query (Table 7, Table S9). Three of these proteins are already known to be involved in vesicle systems of the Arabidopsis cytosol. At1g09180 is described as a Secretion-associated RAS 1 protein (AtSARA1A) GTPase functioning in COPII transport [11], whereas At1g05810 and At4g35860 are listed as Rab proteins, namely the putative RAB-A-class small GTPase (AtRabA5e) and the putative RAB-B-class small GTPase (AtRabB1c), respectively (Table 7).

Chloroplast localization was supported for AtSARA1A and AtRabB1c by both SUBA and Chloroplast 2010 (Table 7). For AtRabA5e, a transit peptide directing the protein to the chloroplast has been previously suggested [14], its chloroplast location — supported by ARAMEMNON and Chloroplast 2010 (Table 7) — was recently confirmed and it was renamed CPRabA5e to better reflect its location [25]. The only one of these four proteins not already recorded as part of the secretory system [11] is the putative GTPase of unknown function/At5g57960, assigned a chloroplast location by ARAMEMNON, supported by SUBA and Chloroplast 2010, rendering it a candidate Arf in chloroplasts (Table 7).

### Putative CCV, COPI and Coat GTPase related chloroplast proteins in rice

In total, 15 proteins in *O. sativa* (subsp. *japonica*) chloroplasts were found to have domains, or combinations of domains, characteristic of CCV, COPI including Coat GTPases (Table 8). Nine of these proteins correspond only to a single subunit. Two, Clathrin heavy chain 1/LOC_Os11g01380 and Clathrin heavy chain 2/LOC_Os12g01390, were identified as possible clathrin heavy chain proteins with a predicted chloroplast location (Table 8). Both have Clathrin propeller repeat (PF01394), Clathrin, heavy-chain linker (PF09268), Clathrin-H-link (PF13838), Region in Clathrin and VPS (PF00637), Clathrin heavy-chain (CHCR) repeat profile (PS50236) domains and an Orn/DAP/Arg decarboxylases family 2 pyridoxal-P attachment site (PS00878) (Table S10).

**Table 8:** Overview of putative chloroplast localized proteins in rice (*subsp. Japonica*) with characteristic domains of CCV and COPI subunit counterparts.

Subunits	Putative chloroplast localized proteins holding domains characteristics for each subunit respectively
**CCV**	
Clathrin heavy chain	Clathrin heavy chain 1 (LOC_Os11g01380), Clathrin heavy chain 2 (LOC_Os12g01390)
AP1 γ subunit	Probable L-ascorbate peroxidase 7 (APX7) (LOC_Os04g35520), Probable L-ascorbate peroxidase 8 (APX8) (LOC_Os02g34810)
AP1 β1 subunit	Armadillo/beta-catenin-like repeat family protein (LOC_Os11g41990)*, Adaptin, putative (LOC_Os01g43630)*, AP-3 complex subunit delta (LOC_Os01g32880)*
AP2 β2 subunit	Armadillo/beta-catenin-like repeat family protein (LOC_Os11g41990)*, Carbamoyl-phosphate synthase large chain (CARB) (LOC_Os01g38970), Acetyl-CoA carboxylase 2 (ACC2) (LOC_Os05g22940), Adaptin, putative (LOC_Os01g43630)*, AP-3 complex subunit delta (LOC_Os01g32880)*
AP2 μ2 subunit	Adaptor complexes medium subunit family protein (LOC_Os12g34370)*
AP3 δ subunit	Armadillo/beta-catenin-like repeat family protein (LOC_Os11g41990)*, Adaptin, putative (LOC_Os01g43630)*, AP-3 complex subunit delta (LOC_Os01g32880)*
AP3 β3 subunit	Armadillo/beta-catenin-like repeat family protein (LOC_Os11g41990)*, Adaptin, putative (LOC_Os01g43630)*, AP-3 complex subunit delta (LOC_Os01g32880)*
AP3 μ3 subunit	Adaptor complexes medium subunit family protein (LOC_Os12g34370)*
AP4 β4 subunit	Adaptin, putative (LOC_Os01g43630)*
AP4 ε subunit	Armadillo/beta-catenin-like repeat family protein (LOC_Os11g41990)*, Adaptin, putative (LOC_Os01g43630)*, AP-3 complex subunit delta (LOC_Os01g32880)*
AP5 μ5 subunit	Adaptor complexes medium subunit family protein (LOC_Os12g34370)*
**COPI**	
B-COPI α-subunit	Guanine nucleotide-binding protein subunit beta (LOC_Os03g46650)*
B-COPI β′-subunit	Guanine nucleotide-binding protein subunit beta (LOC_Os03g46650)*, Regulatory-associated protein of TOR 1 (RAPTOR1) (LOC_Os12g01922)
B-COPI ε-subunit	PPR repeat containing protein (LOC_Os07g14530)
F-COPI δ subunit	Adaptor complexes medium subunit family protein (LOC_Os12g34370)*
F-COPI ζ-subunit	Magnesium-chelatase subunit ChlD (LOC_Os03g59640)
**Coat GTPases**	
ArfA group	Mitochondrial Rho GTPase (LOC_Os03g59590)*
ArfB group	Mitochondrial Rho GTPase (LOC_Os03g59590)*
ArfB2 group	Mitochondrial Rho GTPase (LOC_Os03g59590)*
ArfD group	Mitochondrial Rho GTPase (LOC_Os03g59590)*

* = proteins with several assigned roles, having common occurring domains.

Two other proteins were identified as putative AP1 γ subunits: Probable L-ascorbate peroxidase 7 (APX7)/LOC_Os04g35520 and Probable L-ascorbate peroxidase 8 (APX8)/LOC_Os02g34810 (Table 8), both of which have a Peroxidases proximal heme-ligand signature (PS00435) (Table S10). Two putative AP2 β2 subunits in rice chloroplasts were also identified: Acetyl-CoA carboxylase 2 (ACC2)/LOC_Os05g22940 and Carbamoyl-phosphate synthase large chain (CARB)/LOC_Os01g38970 (Table 8), both containing a Carbamoyl-phosphate synthase subdomain signature 2 domain (PS00867) (Table S10).

Further, a Regulatory-associated protein of TOR 1 (RAPTOR1)/LOC_Os12g01922 was found to have the required domains — a Trp-Asp (WD) repeats profile (PS50082) and a Trp-Asp (WD) repeats circular profile (PS50294) —for a functional B-COPI β′ subunit, whereas PPR repeat-containing protein/LOC_Os07g14530 has the Coatomer epsilon subunit domain (PF04733) required for B-COPI ε subunits (Table 8, Table S10). In addition, Magnesium-chelatase subunit ChlD (CHLD)/LOC_Os03g59640 was found as a putative F-COPI ζ subunit, with a VWFA domain profile (PS50234), in rice chloroplasts (Table 8, Table S10).

In contrast, the remaining six proteins have commonly occurring domains identifying them as possible homologues for several subunits (Table S10). The Adaptin N terminal region (PF01602) domain was found in Armadillo/beta-catenin-like repeat family protein/LOC_Os11g41990, Adaptin, putative/LOC_Os01g43630 and the protein AP3 complex subunit delta/LOC_Os01g32880, identifying them as candidate AP1 β1, AP2 β2, AP3 δ, AP3 β3 and AP4 ε (Table 8, Table S10). In addition, Adaptin, putative/LOC_Os01g43630 has a Beta2-adaptin appendage C-terminal sub-domain (PF09066), providing the domains needed to be a putative AP4 β4 subunit (Table 8, Table S10). Further, Adaptor complexes medium subunit family protein/LOC_Os12g34370 was identified as a putative AP2 μ2, AP3 μ3, AP5 μ5 and F-COPI δ subunit, having Adaptor complexes medium subunit family domain (PF00928), and the Guanine nucleotide-binding protein subunit beta/LOC_Os03g46650 was identified as a possible B-COPI α or B-COPI β′ subunit, with a Trp-Asp (WD) repeats profile PS50082, Trp-Asp (WD) repeats circular profile (PS50294) and Trp-Asp (WD) repeats signature (PS00678). Finally, a candidate for all ARF groups was identified: the Mitochondrial Rho GTPase/LOC_Os03g59590 (Table 8), having an ADP-ribosylation factor family domain (PF00025) (Table 8, Table S10).

## Discussion

We identified 22 proteins in Arabidopsis that may function as parts of a functional COPI or CCV transport system in chloroplasts: a putative clathrin heavy chain component, clathrin light chain, five AP1 γ subunits, five AP2 β2 subunits, eight B-COPI β′ subunits, and two F-COPI ζ subunit proteins (all having characteristic domains, patterns or motifs of cytosolic counterparts; Table 9).

**Table 9:** Overview of putative chloroplast-localized proteins with characteristic domains of CCV and COPI subunit counterparts.

Subunits	Putative chloroplast localized proteins holding domains characteristics for each subunit respectively
**CCV**	
Clathrin heavy chain	AtCHC2
Clathrin light chain	AtCLC1
AP1 γ subunit	Putative ascorbate peroxidase, AttAPX, AtRNEE/G, AtsAPX, AtAPX3
AP1 β1 subunit	Putative gamma subunit of coatomer adaptor complex*, Unknown protein* (At1g51350)
AP2 β2 subunit	AtCarB, Putative H-protein of glycine decarboxylase, AtACC2, AtCAC2, Putative RimM-like protein involved in 16S rRNA processing, Putative gamma subunit of coatomer adaptor complex*, Unknown protein* (At1g51350)
AP2 μ2 subunit	Unknown protein* (At5g57460)
AP3 δ subunit	Putative gamma subunit of coatomer adaptor complex*, Unknown protein* (At1g51350)
AP3 β3 subunit	Putative gamma subunit of coatomer adaptor complex*, Unknown protein* (At1g51350)
AP4 ε subunit	Putative gamma subunit of coatomer adaptor complex*, Unknown protein* (At1g51350)
AP4 μ4 subunit	Unknown protein* (At5g57460)
AP4 σ4 subunit	Unknown protein* (At5g57460)
AP5 μ5 subunit	Unknown protein* (At5g57460)
**COPI**	
B-COPI β′ subunit	AtRACK1A, AtPUB60, AtFZR2/AtCCS52A1, AtFZR3/AtCCS52B, AtMSI1, Unknown proteins (At1g24130, At4g02660, At1g15850)
F-COPI γ subunit	Putative gamma subunit of coatomer adaptor complex*
F-COPI δ subunit	Unknown protein* (At5g57460)
F-COPI ζ subunit	AtCHLD, Unknown protein (At1g67120)
**Coat GTPases**	
ArfA group	AtSARA1A*, AtRabA5e*, AtRabB1c*, Putative GTPase of unknown function*
ArfB group	AtSARA1A*, AtRabA5e*, AtRabB1c*, Putative GTPase of unknown function*
ArfD group	AtSARA1A*, AtRabA5e*, AtRabB1c*, Putative GTPase of unknown function*
ArfB2 group	AtSARA1A*, AtRabA5e*, AtRabB1c*, Putative GTPase of unknown function*

* = proteins with several assigned roles, having common occurring domains.

In addition, seven chloroplast proteins with commonly occurring domains were identified as several putative subunits, possibly with multiple functions (Table 9). Four were identified as similar to Arf proteins, while two others could function as an AP1 β1, AP2 β2, AP3 δ, AP3 β3 and/or AP4 ε subunit. One of these two could also function as an F-COPI γ subunit. The seventh protein was found to have a potential function as an AP2 μ2, AP4 μ4, AP4 σ4, AP5 μ5 and/or F-COPI δ subunit (Table 9).

Thus, various possible components of a COPI or CCV system have been detected in chloroplasts, but several of these have other assigned roles, whereas some required components could not be identified at all. Hence, a key question is whether sufficient components are present to form a functional COPI- or CCV-like transport system.

### Evidence for clathrin-coated vesicle system components in Arabidopsis chloroplasts

#### Triskelion proteins

Concerning triskelion proteins AtCHC2 and AtCLC1 were identified as putative clathrin heavy and light chain respectively inside chloroplast, but have previously been assigned same roles in the cytosol [11], [67]–[69] but both SUBA and Chloroplast 2010 indicate a chloroplast location (Table 1). A possible explanation for this apparent discrepancy, supported by mass spectrometry experiments [70]–[73], is that they have dual locations. However, further tests of this hypothesis are required.

#### AP1

Known Arabidopsis AP1 complex γ subunit proteins have specific domains, or combinations of domains, detected in none of the chloroplast localized proteins (Table S2). However, the AP1 complex γ subunit in yeast contains a domain called Peroxidases proximal heme-ligand signature (PS00435), which was also found in five proteins in the Arabidopsis chloroplast dataset: Putative ascorbate peroxidase, AttAPX, AtsAPX, and AtAPX3 (all ascorbate peroxidases), and the RNAse AtRNEE/G (Table 2). AttAPX, AtRNEE/G, and AtsAPX were predicted to be chloroplast-targeted, but Putative ascorbate peroxidase and AtAPX3 do not have unambiguously chloroplast locations (Table 2). Regardless of the localization it could be argued that even if having the same domain as the yeast AP1 complex γ subunit the proteins are not likely to act as components in the AP complex since they act as peroxidases rather than as clathrin-related components.

AP1 and AP2 complexes in the Arabidopsis cytosol are believed to share a β1/β2 subunit. Since the constitution of a hypothetical COPI or CCV system in the chloroplast is inevitably unknown, we included the separate β1 subunit of AP1 and β2 subunit of AP2 from yeast, in addition to the β1/β2 subunit from Arabidopsis as queries in our searches. As for AP1, this made no difference since we found no chloroplast proteins similar to either β1/β2 or β1 (Table S2).

#### AP2

Regarding the AP2 complex, we found a characteristic domain of the β2 subunit (PS00867) in five chloroplast proteins: Putative H-protein of glycine decarboxylase, AtCarB, AtACC2, AtCAC2, and Putative RimM-like protein involved in 16S rRNA processing (Table S3). This indicates that these proteins are more similar to the yeast subunits than those in the Arabidopsis cytosol. Furthermore, although they have a predicted chloroplast location they have already been assigned roles that are not related to vesicle transport (Figure 2). The Putative H-protein of glycine decarboxylase is a component of the glycine decarboxylase complex (GDC) that decarboxylates and deaminates glycine, a step in photorespiration occurring in mitochondria [74], despite experimental indications of a chloroplast location [72] (Table 3). AtCarB is part of a Carbamoyl phosphate synthase involved in arginine synthesis, likely in the chloroplast [75]. AtACC2 and AtCAC2 are both acetyl-CoA carboxylases (AACs) [76], [77]. The role of AACs is to convert acetyl-CoA to malonyl-CoA during fatty acid synthesis, and plants generally have two types: heterotrimeric and homomeric ACCs. The heterotrimeric ACC in the chloroplast has four subunits: a biotin carboxyl carrier protein, a biotin carboxylase and two carboxyl transferases (α and β) [78]. The homomeric ACC is encoded by two genes, *ACC1* and *ACC2*, and has been considered to be cytosolic, but it was recently shown that the *ACC2* protein product is located in plastids of Arabidopsis [78]. The last putative protein to be discussed as an AP2 β2 subunit is the Putative RimM-like protein involved in 16S rRNA processing, which has been found in the stroma and is involved in RNA processing [70]. Thus, none of the proteins identified as putative AP2 β2 subunits are likely to act in this manner in the chloroplast based on their proposed functions, which are not related to vesicle transport (Figure 2).

In contrast to AP1 and AP2 subunit candidates, putative AP3, AP4 and AP5 subunits identified only had commonly occurring domains, making them weaker candidates as true vesicle transport system components (Figure 2).

### Evidence for COPI vesicle system components in Arabidopsis chloroplasts

#### B-COPI subcomplex

Eight candidate proteins for the B-COPI coat β′ subunit of COPI vesicles with a predicted chloroplast location were detected. The first is AtRACK1 (Table 5), which plays various roles in plants. Plant mutants defective in this protein have reduced sensitivity to various hormones and impairments in developmental processes, including leaf production [79]. AtRACK1A is also a negative regulator of abscisic acid (ABA) responses [80], and recently a number of proteins have been suggested to interact with AtRACK1A, including proteins involved in photosynthesis and stress responses [81]. Its location is ambiguous; chloroplast localization lacks support from ARAMEMNON and Chloroplast 2010, but it has been found experimentally in chloroplasts according to SUBA (Table 5).

The next three identified proteins (AtPUB60, AtFZR2/CCS52SA1 and AtFZR3/CCS52B) have demonstrated roles in protein degradation (Table 5). AtPUB60 is a U-box protein similar to E3 ubiquitin ligases in yeast and humans, involved in plant innate immunity and plant pathogen resistance [82], in addition to its role in the ubiquitin degradation pathway [83]. In Arabidopsis, the cell cycle process is regulated by a number of cyclins, grouped into A, B, D and H cyclins. Some group B cyclins are degraded during mitosis by a specific ubiquitin E3 ligase, known as anaphase promoting complex (APC), following activation by subunits, which include AtFZR2/CCS52A1 and AtFZR3/CCS52B [84], [85]. Further, both of these proteins are involved in endoreduplication, which increases ploidity by inhibiting mitosis [86]. Considering the roles of AtFZR2/CCS52A1 and AtFZR3/CCS52B one might assume a cytosolic location, but ARAMEMNON assigns a chloroplast location, but this has not been experimentally proven according to SUBA.

Further, AtMSI1 was identified as a putative β′ subunit (Table 5). Together with FAS1 and FAS2 this is a member of the Chromatin assembly factor-1 (CAF-1) complex in Arabidopsis, which functions as a histone chaperone in chromatin assembly [87], [88], and is also important for additional processes such as seed development [89].

The last three proteins identified in this category are unknown and largely uncharacterized (Table 5). Two, Unknown proteins At1g15850 and At1g24130 are only known to be nucleotide binding, having WD40 domains. The third (At4g02660), is a putative transport protein with a BEACH domain, found in trichome cells [90] and chloroplasts [71]. The function of the BEACH domain is unknown, but appears to be crucial for a number of proteins involved in e.g. vesicle transport [90], [91]. However this domain could not be identified using Prosite or Pfam. Thus, only the three Unknown proteins can be considered as likely candidates for hypothesized β′ subunits, as the only ones lacking other assigned roles.

#### F-COPI subcomplex

Two proteins were identified as putative ζ subunits of the F-COPI subcomplex: AtCHLD and an Unknown protein (At1g67120) (Table 6). AtCHLD has already been identified as involved in the secretory system [11]. Closer examination revealed that AtCHLD significantly differs from known ζ subunits in Arabidopsis and yeast secretory systems (Table S8). It has a VWFA domain profile (PS50234), similar to von Willebrand factor type A domain (PF13519), and a Magnesium chelatase subunit ChlI domain (PF01078), which are not present in any other known ζ subunits. Other ζ subunits have a Clathrin adaptor complex small chain domain (PF01217) lacked by AtCHLD. Thus, AtCHLD appears to be the Magnesium-chelatase subunit ChlD (Uniprot) of Magnesium chelatase, a complex with three subunits [92], [93]. This complex is involved in chlorophyll biosynthesis, mediating insertion of magnesium ions into protoporphyrin IX, thereby generating Mg-protoporphyrin IX, and is located in chloroplasts [94], [95].

The other putative ζ subunit identified in the chloroplast dataset was the Unknown protein (At1g67120) (Table 6), likely to be chloroplastic according to Chloroplast 2010 and SUBA [73].

Given the distinct differences between AtCHLD and other known ζ subunits, previous reports that AtCHLD functions as a magnesium chelatase in the chloroplast [92], [93], and the finding that the Unknown protein has the same domains as AtCHLD, there are probably no homologues of the ζ subunit in chloroplasts (Figure 2).

### Commonly occurring domains in Arabidopsis chloroplast proteins

Some proteins are reported to perform several roles, such as the AP4 μ4 subunit in Arabidopsis cytosol which has also been noted as the σ4 subunit in the same complex [11] and the newly identified AP5 ζ subunit which has been previously designated a DNA helicase [96]. Thus, the possibility that some of the putative subunits identified here could play several roles and/or other roles than previously reported should not be excluded. We found three proteins that all correspond to several known subunits: Putative gamma subunit of coatomer adaptor complex, and Unknown proteins At1g51350 and At5g57460 (Table 9). The Putative gamma subunit of the coatomer adaptor complex has been ambiguously called both Sec21 and a COPI γ subunit [24], [97]–[99], but is considered to be a γ subunit located in Golgi and ER membranes in Arabidopsis [99]. It has even been used experimentally as a Golgi marker [98], [100] and shown to be involved in cytosolic vesicle transport in Arabidopsis [11], raising doubts about a true chloroplast location, and thus the likelihood of its involvement in vesicle transport in chloroplasts (Figure 2).

It has been suggested that the Unknown protein At1g51350 is a homologue of the human ARMC8α [101], and involved in endosomal sorting and trafficking [102]. However, ARAMEMNON strongly indicates that it is chloroplast localized. The other Unknown protein, At5g57460, has no clear assigned function yet. Thus, the two Unknown proteins could be involved in some of the suggested functions, but further confirmation is needed (Figure 2).

### Coat GTPases in Arabidopsis chloroplasts

Four other proteins with commonly occurring domains were found in the Arabidopsis chloroplast, sharing domains with the previously described cytosolic Arf proteins: AtSARA1A, AtRabA5e, AtRabB1c and the Putative GTPase of unknown function (Table 7). All but one of these four proteins has been ascribed other functions, showing that searches for proteins with this domain will not detect only Arf proteins. The Putative GTPase of unknown function, strongly predicted to be chloroplastic by all the databases and experimental data [70], [71], is downregulated in a cold-resistant *bri1* (brassinosteroid-insensitive 1) Arabidopsis mutant [103]. Thus, its possible involvement in vesicle transport is not clear (Figure 2).

AtSARA1A has both a small GTPase Arf family profile domain (PS51417) and an ADP-ribosylation factor family domain (PF00025) and has already been identified in the secretory system of Arabidopsis as a Sar1 protein [11] (Table S9). It acts as a GTPase, regulating COPII coat assembly in the cytosol [104], [105]. However, SARA1A has also been detected in chloroplasts [106], thus it has an ambiguous or possibly dual localization. Interestingly, another Sar1 protein, CPSAR1, identified in the chloroplast has been shown to affect vesicle transport [9].

Two Rab proteins were identified, CPRabA5e and RabB1c (Table 7). CPRabA5E has previously been predicted as an Arf protein [24], but was recently shown to be a Rab protein with a chloroplast location involved in thylakoid biogenesis. It was affected by oxidative stress, accumulating vesicles at the envelope in chloroplasts when incubated at low temperature under oxidative stress [25].

RabB1c is assumed to participate in vesicle transport according to Uniprot, and is a member of the AtRabB family, which is related to human Rab2 GTPases that are involved in COPI transport in mammalian cells [107], [108], and may play a similar role in the Arabidopsis secretory system [109]. RabB1c lacks a transit peptide [14], but has been detected in chloroplasts experimentally [71]. Thus, the only plausible candidate Arf in the chloroplast is the Putative GTPase of unknown function, but GTP binding is apparently not sufficient for a functional Arf, thus further confirmation that it acts as one is required (Figure 2).

### Evidence for clathrin-coated vesicle system components in rice

Six proteins were identified in rice with CCV relevant domains and chloroplast localization according to Target P. Clathrin heavy chain 1 and Clathrin heavy chain 2 (Table 8) are referred to as clathrin heavy chains [110], [111], based on their similarity to other clathrin components (Uniprot) but have not been characterized. If the predicted chloroplast localization is correct further investigation is warranted since their homologies and designations clearly imply a role in vesicle transport.

Two ascorbate peroxidases were identified, APX7 and APX 8 (Table 8). In plants, ascorbate peroxidases use ascorbate as an electron donor to convert H_2_O_2_ to H_2_O. In rice there are eight known *APX* genes, and four of which are believed to be chloroplast localized (*APX5-APX8*) [112], [113]. However, they are unlikely to act as AP1 γ subunits in chloroplasts due to their role as peroxidases.

Two AP2 β2 subunit candidates, ACC2 and CARB (Table 8), were identified. However, as discussed above in the Arabidopsis analysis, ACC2 and CARB are involved in fatty acid and arginine synthesis; hence they are unlikely to be subunits of chloroplast vesicles.

### Evidence for COPI system components in rice

With COPI relevant domains and chloroplast location according to Target P, three proteins were identified. RAPTOR1 was identified as a putative B-COPI β′ subunit (Table 8). However, in Arabidopsis RAPTOR1 is known as to regulate TOR1 (TARGET OF RAPAMYCIN), a kinase involved in growth signalling pathways, and interacts with a putative substrate of TOR, S6K1, in vivo [114]. The role of RAPTOR1 in the TOR pathway in Arabidopsis makes it an unlikely candidate as possible B-COPI β′ subunit also in rice.

As a putative B-COPI ε subunit, the PPR repeat containing protein was identified (Table 8). Pentatricopeptide repeat proteins (PPR proteins) are RNA-binding proteins involved in various post-transcriptional processes in both mitochondria and chloroplasts [115]. The PPR family is defined by a tandem 35 amino acid motif. The proteins are predicted to have multiple α helices, placing them in the α-solenoid superfamily together with e.g. HEAT domain proteins [115], [116]. One of the PPR proteins in rice, OsPPR1, is located in chloroplasts, essential for chloroplast biogenesis, and its suppression results in chlorophyll deficiency [117].

As also found in the Arabidopsis analysis, the only protein corresponding to F-COPI ζ identified in rice chloroplasts was a magnesium chelatase, CHLD [118] (Table 8). This again raises doubts about its function as a COPI component, which was previously indicated [11].

### Commonly occurring domains of proteins including Coat GTPases in rice chloroplasts

Six proteins of rice chloroplasts were identified with commonly occurring domains found in multiple subunits, but due to the low specificity of the identifying domains they are less robust candidates. One domain, the Adaptin N terminal region (PF01602), was detected in AP-3 complex subunit delta, Armadillo/beta-catenin-like repeat family protein and Adaptin, putative (Table S10). Little is known about these proteins; AP-3 complex subunit delta has a name implying a role in vesicle transport, but has not yet been characterized. The Armadillo/beta-catenin-like repeat family protein has Armadillo repeats, placing it in the ARM repeat superfamily together with AP-3 complex subunit delta, according to Uniprot and Superfamily 1.75 [119]. Armadillo repeats are found in proteins with various roles, for instance β-catenin [120]. They are about 40 amino acids long and usually tandemly repeated, forming an armadillo domain. Adaptin, putative has an Adaptin N terminal region (PF01602), but also a Beta2-adaptin appendage C-terminal sub-domain (PF09066) (Table S10). The protein has not yet been characterized in rice. Since two of these proteins have names related to vesicle transport, and all three share domain PF01602, they could potentially all be true subunits.

Adaptor complexes medium subunit family protein/LOC_Os12g34370 has, similarly to AP-3 complex subunit delta and Adaptin putative not either been characterized but a name implying a role in vesicle transport. In Arabidopsis, At1g56590 was annotated as Clathrin adaptor complexes medium subunit family protein [121] and is considered as the AP3 μ3 subunit [11]. Hence, a role in vesicle transport in chloroplasts cannot be excluded.

Guanine nucleotide-binding protein subunit beta was identified as a putative B-COPI α and B-COPI β′ subunit. Its name implies a role as a subunit of a heterotrimeric G-protein, but it has not yet been characterized. The domains identified in this protein (PS50294, PS50082 and PS00678) all refer to WD repeats (Table S10). WD repeat proteins have four of more repetitive subunits, each consisting of about 40–60 amino acids and usually ending with tryptophan (W) and aspartic acid (D) [122]. It has been assumed that all WD repeat proteins form β propellers, and the best characterized is the β subunit of the heterotrimeric G protein [39], [122], [123]. WD repeat proteins have known importance in various processes, including vesicle transport [122], [123], but as shown here simply detecting WD repeats in a protein is not sufficient to elucidate a protein's functions completely.

The Ras superfamily of small GTPases is divided into five families: Rab, Arf/Sar, Ran, Ras and Rho. In plants, no representatives of the Ras family have been found [124]. One protein was identified in a search for proteins with an ADP-ribosylation factor family domain (PF00025): Mitochondrial Rho GTPase. Its Uniprot name indicates a mitochondrial location, but its Rice Genome Annotation project designation is less specific (ATP/GTP/Ca++ binding protein, putative, expressed), and it is located in the chloroplast according to Target P (Table S10). Thus, future experiments are needed to resolve its location.

## Conclusion

The acquired data indicate that no transport system resembling cytosolic CCV or COPI systems is present in Arabidopsis chloroplasts. Several putative subunits identified in the chloroplast dataset were shown to be located elsewhere according to previous studies or various tools, having a possible dual location and/or roles unrelated to vesicle transport. Out of 29 proteins identified in Arabidopsis, the majority had either commonly occurring domains, vesicle unrelated or unknown function (Figure 2). Only two proteins among the suggested, Putative heavy chain of clathrin complex (AtCHC2)/At3g08530 and Putative light chain of clathrin complex (AtCLC1)/At2g40060, could be considered likely subunits in the chloroplast, having known roles related to vesicle transport. Several subunits could not be identified at all in the chloroplast, when searching for relevant domains (Figure 2). The findings indicate that if a CCV- or COPI-like vesicle system is present in chloroplasts it probably differs substantially from the cytosolic counterpart. However, the possible presence of a different and/or simplified CCV or COPI system cannot be excluded. The occurrence of a putative AP2 β2 subunit supports the possible presence of a unique system, since this homologue is present in yeast but not Arabidopsis cytosol, and many of the putative subunits identified have greater resemblance to yeast counterparts than Arabidopsis counterparts (Tables S1, S2, S3, S4, S5, S6, S7, S8, S9).

Considering rice, most of the subunits that could be identified are uncharacterized and named by their similarity to other proteins. As in Arabidopsis chloroplasts, many proteins were also found to have commonly occurring domains. Only two proteins (still uncharacterized in rice) have names indicating a role in vesicle transport, a predicted chloroplast location and domains that are not commonly occurring: Clathrin heavy chain 1 and Clathrin heavy chain 2. It is interesting to note that the results in rice support the findings in Arabidopsis i.e. not many proteins can be clearly said to be chloroplast localized and involved in vesicle transport.

No prokaryote vesicle transport system has been reported [16], [125], [126], but a few examples of prokaryotic structures analogous to vesicles have been observed [35]. The vesicle system in eukaryotes has been hypothesized as a trait that developed soon after the divergence from prokaryotes and thereafter further specialized as adaptations to new environments [35]. Chloroplasts, believed to have resulted via endosymbiosis of early eukaryotes with cyanobacteria, have vesicles with properties resembling other eukaryotic vesicles, including probable regulation of their formation by GTPases, and inhibition of fusion by microcystin LR and low temperature [16]. However, two proteins of prokaryotic origin have suggested involvement in vesicle formation in chloroplasts: CPSAR1 and Vipp1 [9], [127]. Vesicles have also been found in representatives of embryophytes, including bryophytes, pteridophytes, spermatophytes (gymnosperms and angiosperms), but not in other groups including cyanobacteria, glaucocystophytes, rhodophytes, chlorophytes and charophytes. Hence, it has been proposed that the vesicles in chloroplasts evolved after the division of embryophytes from charophytes as an adaptation to land colonization [125].

Plastids occur in several forms, in diverse organisms, and their broad variation in thylakoid organization is assumed to have arisen via evolution in different hosts after the ancestral endosymbiosis [126]. Regarding the three known vesicle transport systems, the COPII system is likely to ancestral, since it is used in essential biosynthetic pathways in all eukaryotes, while the COPI and CCV systems could be later specializations involved in recycling resources to the ER and endocytosis [26], [128], [129].

Taken together, the available evidence indicates that a vesicle system arose in early eukaryotes, COPII is the ancestral machinery, chloroplast vesicles show clear eukaryotic traits and first evolved during land colonization. In addition, we conclude that no COPI- or CCV-like vesicle system is likely to be found in chloroplasts, in contrast to a COPII-like system, for which a chloroplast location has bioinformatic support [14]. Speculatively, early eukaryotes gained a COPII-like vesicle system, engulfed cyanobacteria and developed plastids, to which the system was transferred. If so, since some photosynthetic eukaryotes do not have vesicles in their plastids, a major speciation event was presumably involved, separating those that form COPII-like chloroplast vesicles from others, before all lines continued to develop the COPI and CCV systems in the cytosol. Alternatively, all three vesicle systems may have already developed in the cytosol of the ancestral eukaryotes when cyanobacteria were engulfed, but only the COPII system was transferred to the chloroplast, or the other two were lost during subsequent evolution. Thus, future experimental evidence is needed to solve the intriguing questions how, when and why a suggested COPII system emerged as the sole vesicle system in chloroplasts.

## Supporting Information

Table S1 CCV triskelion proteins from Arabidopsis (*A. thaliana*) cytosol (retrieved from Bassham et al, 2008) and yeast (*S. cerevisiae*), mouse (*M. musculus*) and human (*H. sapiens*) cytosol (retrieved from Uniprot). Domains of these proteins were extracted using Prosite and Pfam, then run against the chloroplast protein dataset to identify proteins putatively involved in vesicle transport in chloroplasts.
(PDF)

Table S2 CCV AP1 complex proteins from Arabidopsis (*A. thaliana*) cytosol (retrieved from Bassham et al, 2008) and yeast (*S. cerevisiae*), mouse (*M. musculus*) and human (*H. sapiens*) cytosol (retrieved from Uniprot). Domains of these proteins were extracted using Prosite and Pfam, then run against the chloroplast protein dataset to identify proteins putatively involved in vesicle transport in chloroplasts.
(PDF)

Table S3 CCV AP2 complex proteins from Arabidopsis (*A. thaliana*) cytosol (retrieved from Bassham et al, 2008) and yeast (*S. cerevisiae*), mouse (*M. musculus*) and human (*H. sapiens*) cytosol (retrieved from Uniprot). Domains of these proteins were extracted using Prosite and Pfam, then run against the chloroplast protein dataset to identify proteins putatively involved in vesicle transport inside chloroplasts.
(PDF)

Table S4 CCV AP3 complex proteins from Arabidopsis (*A. thaliana*) cytosol (retrieved from Bassham et al, 2008) and yeast (*S. cerevisiae*), mouse (*M. musculus*) and human (*H. sapiens*) cytosol (retrieved from Uniprot). Domains of these proteins were extracted using Prosite and Pfam, then run against the chloroplast protein dataset to identify proteins putatively involved in vesicle transport inside chloroplasts.
(PDF)

Table S5 CCV AP4 complex proteins from Arabidopsis (*A. thaliana*) cytosol (retrieved from Bassham et al, 2008) and yeast (*S. cerevisiae*), mouse (*M. musculus*) and human (*H. sapiens*) cytosol (retrieved from Uniprot). Domains of these proteins were extracted using Prosite and Pfam, then run against the chloroplast protein dataset to identify proteins putatively involved in vesicle transport in chloroplasts.
(PDF)

Table S6 CCV AP5 complex proteins from Arabidopsis (*A. thaliana*) cytosol (retrieved from Hirst et al, 2011), and human (*H. sapiens*) cytosol (retrieved from Uniprot). Domains of these proteins were extracted using Prosite and Pfam, then run against the chloroplast protein dataset to identify proteins putatively involved in vesicle transport inside chloroplasts.
(PDF)

Table S7 B-COPI subcomplex proteins from Arabidopsis (*A. thaliana*) cytosol (retrieved from Bassham et al, 2008) and yeast (*S. cerevisiae*), mouse (*M. musculus*) and human (*H. sapiens*) cytosol (retrieved from Uniprot). Domains of these proteins were extracted using Prosite and Pfam, then run against the chloroplast protein dataset to identify proteins putatively involved in vesicle transport inside chloroplasts.
(PDF)

Table S8 F-COPI subcomplex proteins from Arabidopsis (*A. thaliana*) cytosol (retrieved from Bassham et al, 2008) and yeast (*S. cerevisiae*), mouse (*M. musculus*) and human (*H. sapiens*) cytosol (retrieved from Uniprot). Domains of these proteins were extracted using Prosite and Pfam, then run against the chloroplast protein dataset to identify proteins putatively involved in vesicle transport inside chloroplasts.
(PDF)

Table S9 Coat GTPase proteins from Arabidopsis (*A. thaliana*) cytosol (retrieved from Bassham et al, 2008) and yeast (*S. cerevisiae*), mouse (*M. musculus*) and human (*H. sapiens*) cytosol (retrieved from Uniprot). Domains of these proteins were extracted using Prosite and Pfam, then run against the chloroplast protein dataset to identify proteins putatively involved in vesicle transport inside chloroplasts.
(PDF)

Table S10 CCV and COPI proteins from Arabidopsis (*A. thaliana*) cytosol (retrieved from Bassham et al, 2008) and yeast (*S. cerevisiae*), mouse (*M. musculus*) and human (*H. sapiens*) cytosol (retrieved from Uniprot). Domains of these proteins were extracted using Prosite and Pfam, run against the rice (subsp. *japonica*) protein dataset to identify proteins with the same domains, then those putatively involved in vesicle transport in chloroplasts were identified using Target P, and listed.
(PDF)
