# Supplementary material for: Bioinformatic Indications That COPI- and Clathrin-Based Transport Systems Are Not Present in Chloroplasts: An Arabidopsis Model
Source: PLoS One. 2014 Aug 19;9(8):e104423. doi: 10.1371/journal.pone.0104423 (PMC4138088; doi:10.1371/journal.pone.0104423)
Supplement: Table S1 — CCV triskelion proteins from Arabidopsis (A. thaliana) cytosol (retrieved from Bassham et al, 2008) and yeast (S. cerevisiae), mouse (M. musculus) and human (H. sapiens) cytosol (retrieved from Uniprot). Domains of these proteins were extracted using Prosite and Pfam, then run against the chloroplast protein dataset to identify proteins putatively involved in vesicle transport in chloroplasts. (PDF) [file pone.0104423.s001.pdf]

Table S1. CCV triskelion proteins from Arabidopsis (*A. thaliana*) cytosol (retrieved from Bassham et al, 2008) and yeast (*S. cerevisiae*), mouse (*M. musculus*) and human (*H. sapiens*) cytosol (retrieved from Uniprot). Domains of these proteins were extracted using Prosite and Pfam, then run against the chloroplast protein dataset to identify proteins putatively involved in vesicle transport in chloroplasts.

**Organism, Accession No., Uniprot ID**	**Prosite profile/ pattern, Entry No.**	**Chloroplast proteins, Prosite Entry No.**	**Pfam profile/pattern, Entry No.**	**Chloroplast proteins, Pfam Entry No.**
**Clathrin heavy chain**				
A. thaliana, At3g11130, Q0WNJ6	Clathrin heavy-chain (CHCR) repeat profile: PS50236<br><br>Orn/DAP/Arg decarboxylases family 2 pyridoxal-P attachment site: PS00878	PS50236: At3g08530<br><br>PS00878: n.d.	Clathrin propeller repeat: PF01394<br><br>Clathrin, heavy-chain linker: PF09268<br><br>Clathrin-H-link: PF13838<br><br>Region in Clathrin and VPS: PF00637	PF01394: At3g08530<br><br>PF09268: At3g08530<br><br>PF13838: At3g08530<br><br>PF00637: At3g08530, At3g23020, At5g42310, At2g18940, At5g39980, At3g04760, At2g31400, At2g02150
A thaliana, At3g08530, Q0WLB5	Clathrin heavy-chain (CHCR) repeat profile: PS50236<br><br>Orn/DAP/Arg decarboxylases family 2 pyridoxal-P attachment site: PS00878	PS50236: At3g08530<br><br>PS00878: n.d.	Clathrin propeller repeat: PF01394<br><br>Clathrin, heavy-chain linker: PF09268<br><br>Clathrin-H-link: PF13838<br><br>Region in Clathrin and VPS: PF00637	PF01394: At3g08530<br><br>PF09268: At3g08530<br><br>PF13838: At3g08530<br><br>PF00637: At3g08530, At3g23020, At5g42310, At2g18940, At5g39980, At3g04760, At2g31400, At2g02150
S. cerevisiae, CHC1 YGL206C, P22137	Clathrin heavy-chain (CHCR) repeat profile: PS50236	PS50236: At3g08530	Clathrin, heavy-chain linker: PF09268<br><br>Clathrin-H-link: PF13838<br><br>Region in Clathrin and VPS: PF00637	PF09268: At3g08530<br><br>PF13838: At3g08530<br><br>PF00637: At3g08530, At3g23020, At5g42310, At2g18940, At5g39980, At3g04760, At2g31400, At2g02150
**Clathrin light chain**				
A. thaliana, At2g40060, O04209	n.d.	-	Clathrin light chain: PF01086	PF01086: At2g40060
A. thaliana, At3g51890, F4J5M9	n.d.	-	Clathrin light chain: PF01086	PF01086: At2g40060
S. cerevisiae, CLC1 YGR167W, P17891	Clathrin light chain signature 2: PS00581	PS00581: n.d.	Clathrin light chain: PF01086	PF01086: At2g40060

n.d., not detected
