# Supplementary material for: Bioinformatic Indications That COPI- and Clathrin-Based Transport Systems Are Not Present in Chloroplasts: An Arabidopsis Model
Source: PLoS One. 2014 Aug 19;9(8):e104423. doi: 10.1371/journal.pone.0104423 (PMC4138088; doi:10.1371/journal.pone.0104423)
Supplement: Table S2 — CCV AP1 complex proteins from Arabidopsis (A. thaliana) cytosol (retrieved from Bassham et al, 2008) and yeast (S. cerevisiae), mouse (M. musculus) and human (H. sapiens) cytosol (retrieved from Uniprot). Domains of these proteins were extracted using Prosite and Pfam, then run against the chloroplast protein dataset to identify proteins putatively involved in vesicle transport in chloroplasts. (PDF) [file pone.0104423.s002.pdf]

Table S2. CCV AP1 complex proteins from Arabidopsis (*A. thaliana*) cytosol (retrieved from Bassham et al, 2008), yeast (*S. cerevisiae*), mouse (*M. musculus*) and human (*H. sapiens*) cytosol (retrieved from Uniprot). Domains of these proteins were extracted using Prosite and Pfam, then run against the chloroplast protein dataset to identify proteins putatively involved in vesicle transport inside chloroplasts.

**Organism, Accession No., Uniprot ID**	**Prosite profile/ pattern, Entry No.**	**Chloroplast proteins, Prosite Entry No.**	**Pfam profile/pattern, Entry No.**	**Chloroplast proteins, Pfam Entry No.**
**γ subunit**				
A. thaliana, At1g60070, Q9ZUI6	Gamma-adaptin ear (GAE) domain profile: PS50180	PS50180: n.d.	Adaptin N terminal region: PF01602<br><br>Adaptin C-terminal domain: PF02883	PF01602: At4g34450, At1g51350<br><br>PF02883: n.d.
A. thaliana, At1g23900, Q84K16	Gamma-adaptin ear (GAE) domain profile: PS50180	PS50180: n.d.	Adaptin N terminal region: PF01602<br><br>Adaptin C-terminal domain: PF02883	PF01602: At4g34450, At1g51350<br><br>PF02883: n.d.
S. cerevisiae, APL4 YPR029C, Q12028	Peroxidases proximal heme-ligand signature: PS00435	PS00435: At1g07890, At1g77490, At2g04270, At4g08390, At4g35000	Adaptin N terminal region: PF01602<br><br>Adaptin C-terminal domain: PF02883	PF01602: At4g34450, At1g51350<br><br>PF02883: n.d.
**β1 subunit***				
S. cerevisiae, APL2 YKL135C, P36000	n.d.	-	Adaptin N terminal region: PF01602	PF01602: At4g34450, At1g51350
**β1/β2 subunit**				
A. thaliana, At4g11380, Q9SUS3	n.d.	-	Adaptin N terminal region: PF01602<br><br>Adaptin C-terminal domain: PF02883<br><br>Beta2-adaptin appendage, C-terminal sub-domain: PF09066	PF01602: At4g34450, At1g51350<br><br>PF02883: n.d.<br><br>PF09066: n.d.
A. thaliana, At4g23460, O81742	n.d.	-	Adaptin N terminal region: PF01602	PF01602: At4g34450, At1g51350<br><br>PF02883: n.d.

			Adaptin C-terminal domain: PF02883<br><br>Beta2-adaptin appendage, C-terminal sub-domain: PF09066	PF09066: n.d.
**µ1 subunit**				
A. thaliana, At1g60780, O22715	Mu homology domain (MHD) profile: PS51072<br><br>Clathrin adaptor complexes medium chain signature 1: PS00990<br><br>Clathrin adaptor complexes medium chain signature 2: PS00991	PS51072: At5g57460<br><br>PS00990: n.d.<br><br>PS00991: n.d.	Clathrin adaptor complex small chain: PF01217<br><br>Adaptor complexes medium subunit family: PF00928	PF01217: n.d.<br><br>PF00928: n.d.
A. thaliana, At1g10730, Q9SAC9	Mu homology domain (MHD) profile: PS51072<br><br>Clathrin adaptor complexes medium chain signature 2: PS00991	PS51072: At5g57460<br><br>PS00991: n.d.	Clathrin adaptor complex small chain: PF01217<br><br>Adaptor complexes medium subunit family: PF00928	PF01217: n.d.<br><br>PF00928: n.d.
S. cerevisiae, APM1 YPL259C, Q00776	Mu homology domain (MHD) profile: PS51072<br><br>Clathrin adaptor complexes medium chain signature 1: PS00990<br><br>Clathrin adaptor complexes medium chain signature 2: PS00991	PS51072: At5g57460<br><br>PS00990: n.d.<br><br>PS00991: n.d.	Clathrin adaptor complex small chain: PF01217<br><br>Adaptor complexes medium subunit family: PF00928	PF01217: n.d.<br><br>PF00928: n.d.
**σ1 subunit**				
A. thaliana, At2g17380, Q8LEZ8	Clathrin adaptor complexes small chain signature: PS00989	PS00989: n.d.	Clathrin adaptor complex small chain: PF01217	PF01217: n.d.
A. thaliana, At4g35410,	Clathrin adaptor complexes small	PS00989: n.d.	Clathrin adaptor complex small	PF01217: n.d.

O23685	chain signature: PS00989		chain: PF01217	
M. musculus, Aps1s1, P61967	Clathrin adaptor complexes small chain signature: PS00989	PS00989: n.d.	Clathrin adaptor complex small chain: PF01217	PF01217: n.d.

n.d., not detected
