# Supplementary material for: Bioinformatic Indications That COPI- and Clathrin-Based Transport Systems Are Not Present in Chloroplasts: An Arabidopsis Model
Source: PLoS One. 2014 Aug 19;9(8):e104423. doi: 10.1371/journal.pone.0104423 (PMC4138088; doi:10.1371/journal.pone.0104423)
Supplement: Table S3 — CCV AP2 complex proteins from Arabidopsis (A. thaliana) cytosol (retrieved from Bassham et al, 2008) and yeast (S. cerevisiae), mouse (M. musculus) and human (H. sapiens) cytosol (retrieved from Uniprot). Domains of these proteins were extracted using Prosite and Pfam, then run against the chloroplast protein dataset to identify proteins putatively involved in vesicle transport inside chloroplasts. (PDF) [file pone.0104423.s003.pdf]

Table S3. CCV AP2 complex proteins from Arabidopsis (*A. thaliana*) cytosol (retrieved from Bassham et al, 2008) and yeast (*S. cerevisiae*), mouse (*M. musculus*) and human (*H. sapiens*) cytosol (retrieved from Uniprot). Domains of these proteins were extracted using Prosite and Pfam, then run against the chloroplast protein dataset to identify proteins putatively involved in vesicle transport inside chloroplasts.

Organism, Accession No., Uniprot ID	Prosite profile/ pattern, Entry No.	Chloroplast proteins, Prosite Entry No.	Pfam profile/pattern, Entry No.	Chloroplast proteins, Pfam Entry No.
**α subunit**				
A. thaliana, At5g22770, Q8LPL6	n.d.	-	Adaptin N terminal region: PF01602<br><br>Adaptin C-terminal domain: PF02883<br><br>Alpha adaptin AP2, C-terminal domain: PF02296	PF01602: At4g34450, At1g51350<br><br>PF02883: n.d.<br><br>PF02296: n.d.
A. thaliana, At5g22780, Q8LPK4	n.d.	-	Adaptin N terminal region: PF01602<br><br>Adaptin C-terminal domain: PF02883<br><br>Alpha adaptin AP2, C-terminal domain: PF02296	PF01602: At4g34450, At1g51350<br><br>PF02883: n.d.<br><br>PF02296: n.d.
S. cerevisiae, APL3 YBL037W, P38065	n.d.	-	Adaptin N terminal region: PF01602<br><br>Adaptin C-terminal domain: PF02883	PF01602: At4g34450, At1g51350<br><br>PF02883: n.d.
**β2 subunit***				
S. cerevisiae, APL1 YJR005W, P27351	Carbamoyl-phosphate synthase subdomain signature 2: PS00867	PS00867: At1g29900, At1g32470, At1g36180, At5g35360, At5g46420	Adaptin N terminal region: PF01602	PF01602: At4g34450, At1g51350
**β1/β2 subunit**	See above	See above	See above	See above
**µ2 subunit**				
A. thaliana, At5g46630, B9DI54	Mu homology domain (MHD) profile: PS51072	PS51072: At5g57460	Adaptor complexes medium subunit	PF00928: n.d.

			family: PF00928	
S. cerevisiae, APM4 YOL062C, Q99186	Mu homology domain (MHD) profile: PS51072	PS51072: At5g57460	Adaptor complexes medium subunit family: PF00928	PF00928: n.d.
**σ2 subunit**				
A. thaliana, At1g47830, Q84WL9	n.d.	-	Clathrin adaptor complex small chain: PF01217	PF01217: n.d.
S. cerevisiae, APS2 YJR058C, Q00381	Clathrin adaptor complexes small chain signature: PS00989	n.d.	Clathrin adaptor complex small chain: PF01217	PF01217: n.d.

n.d., not detected
