# Supplementary material for: Bioinformatic Indications That COPI- and Clathrin-Based Transport Systems Are Not Present in Chloroplasts: An Arabidopsis Model
Source: PLoS One. 2014 Aug 19;9(8):e104423. doi: 10.1371/journal.pone.0104423 (PMC4138088; doi:10.1371/journal.pone.0104423)
Supplement: Table S4 — CCV AP3 complex proteins from Arabidopsis (A. thaliana) cytosol (retrieved from Bassham et al, 2008) and yeast (S. cerevisiae), mouse (M. musculus) and human (H. sapiens) cytosol (retrieved from Uniprot). Domains of these proteins were extracted using Prosite and Pfam, then run against the chloroplast protein dataset to identify proteins putatively involved in vesicle transport inside chloroplasts. (PDF) [file pone.0104423.s004.pdf]

Table S4. CCV AP3 complex proteins from Arabidopsis (*A. thaliana*) cytosol (retrieved from Bassham et al, 2008) and yeast (*S. cerevisiae*), mouse (*M. musculus*) and human (*H. sapiens*) cytosol (retrieved from Uniprot). Domains of these proteins were extracted using Prosite and Pfam, then run against the chloroplast protein dataset to identify proteins putatively involved in vesicle transport inside chloroplasts.

Organism, Accession No., Uniprot ID	Prosite profile/ pattern, Entry No.	Chloroplast proteins, Prosite Entry No.	Pfam profile/pattern, Entry No.	Chloroplast proteins, Pfam Entry No.
**δ subunit**				
A. thaliana, At1g48760, Q9C744	n.d.	-	Adaptin N terminal region: PF01602	PF01602: At4g34450, At1g51350
S. cerevisiae, APL5 YPL195W, Q08951	n.d.	-	Adaptin N terminal region: PF01602	PF01602: At4g34450, At1g51350
**β3 subunit**				
A. thaliana, At3g55480, Q9M2T1	n.d.	-	Adaptin N terminal region: PF01602	PF01602: At4g34450, At1g51350
S. cerevisiae, APL6 YGR261C, P46682	n.d.	-	Adaptin N terminal region: PF01602	PF01602: At4g34450, At1g51350
**µ3 subunit**				
A. thaliana, At1g56590, Q8LPJ0	Mu homology domain (MHD) profile: PS51072<br><br>Clathrin adaptor complexes medium chain signature 1: PS00990	PS51072: At5g57460<br><br>PS00990: n.d.	Adaptor complexes medium subunit family: PF00928	PF00928: n.d.
S. cerevisiae, APM3 YBR288C, P38153	Mu homology domain (MHD) profile: PS51072<br><br>Clathrin adaptor complexes medium chain signature 1: PS00990<br><br>Clathrin adaptor complexes medium chain signature 2: PS00991	PS51072: At5g57460<br><br>PS00990: n.d.<br><br>PS00991: n.d.	Adaptor complexes medium subunit family: PF00928	PF00928: n.d.
**σ3 subunit**				
A. thaliana, At3g50860, Q8VZ37	Clathrin adaptor complexes small chain signature: PS00989	PS00989: n.d.	Clathrin adaptor complex small chain: PF01217	PF01217: n.d.

S. cerevisiae, APS3 YJL024C, P47064	Clathrin adaptor complexes small chain signature: PS00989	PS00989: n.d.	Clathrin adaptor complex small chain: PF01217	PF01217: n.d.

n.d., not detected
