# Supplementary material for: Bioinformatic Indications That COPI- and Clathrin-Based Transport Systems Are Not Present in Chloroplasts: An Arabidopsis Model
Source: PLoS One. 2014 Aug 19;9(8):e104423. doi: 10.1371/journal.pone.0104423 (PMC4138088; doi:10.1371/journal.pone.0104423)
Supplement: Table S5 — CCV AP4 complex proteins from Arabidopsis (A. thaliana) cytosol (retrieved from Bassham et al, 2008) and yeast (S. cerevisiae), mouse (M. musculus) and human (H. sapiens) cytosol (retrieved from Uniprot). Domains of these proteins were extracted using Prosite and Pfam, then run against the chloroplast protein dataset to identify proteins putatively involved in vesicle transport in chloroplasts. (PDF) [file pone.0104423.s005.pdf]

Table S5. CCV AP4 complex proteins from Arabidopsis (*A. thaliana*) cytosol (retrieved from Bassham et al, 2008) and yeast (*S. cerevisiae*), mouse (*M. musculus*) and human (*H. sapiens*) cytosol (retrieved from Uniprot). Domains of these proteins were extracted using Prosite and Pfam, then run against the chloroplast protein dataset to identify proteins putatively involved in vesicle transport in chloroplasts.

Organism, Accession No., Uniprot ID	Prosite profile/ pattern, Entry No.	Chloroplast proteins, Prosite Entry No.	Pfam profile/pattern, Entry No.	Chloroplast proteins, Pfam Entry No.
**ε subunit**				
A. thaliana, At1g31730, Q8L7A9	n.d.	-	Adaptin N terminal region: PF01602	PF01602: At4g34450, At1g51350
H. sapiens, AP4E1, Q9UPM8	n.d.	-	Adaptin N terminal region: PF01602<br><br>Adaptin AP4 complex epsilon appendage platform: PF14807	PF01602: At4g34450, At1g51350<br><br>PF14807: n.d.
**β4 subunit**				
A. thaliana, At5g11490, Q9LDK9	n.d.	-	Adaptin N terminal region: PF01602<br><br>Beta2-adaptin appendage, C-terminal sub-domain: PF09066	PF01602: At4g34450, At1g51350<br><br>PF09066: n.d.
M. musculus, Ap4b1, Q9WV76	n.d.	-	Adaptin N terminal region: PF01602<br><br>Beta2-adaptin appendage, C-terminal sub-domain: PF09066	PF01602: At4g34450, At1g51350<br><br>PF09066: n.d.
**μ4 subunit**				
A. thaliana, At4g24550, Q9SB50	Mu homology domain (MHD) profile: PS51072	PS51072: At5g57460	Clathrin adaptor complex small chain: PF01217<br><br>Adaptor complexes medium subunit family: PF00928	PF01217: n.d.<br><br>PF00928: n.d.
H. sapiens, AP4M1, O00189	Mu homology domain (MHD) profile: PS51072	PS51072: At5g57460<br><br>PS00991: n.d.	Clathrin adaptor complex small chain: PF01217	PF01217: n.d.<br><br>PF00928: n.d.

	Clathrin adaptor complexes medium chain signature 2: PS00991		Adaptor complexes medium subunit family: PF00928	
**σ4 subunit**				
A. thaliana, At4g24550, Q9SB50	Mu homology domain (MHD) profile: PS51072	PS51072: At5g57460	Clathrin adaptor complex small chain: PF01217<br><br>Adaptor complexes medium subunit family: PF00928	PF01217: n.d.<br><br>PF00928: n.d.
H. sapiens, AP4S1, Q9Y587	n.d.	-	Clathrin adaptor complex small chain: PF01217	PF01217: n.d.

n.d., not detected
