# Supplementary material for: Bioinformatic Indications That COPI- and Clathrin-Based Transport Systems Are Not Present in Chloroplasts: An Arabidopsis Model
Source: PLoS One. 2014 Aug 19;9(8):e104423. doi: 10.1371/journal.pone.0104423 (PMC4138088; doi:10.1371/journal.pone.0104423)
Supplement: Table S6 — CCV AP5 complex proteins from Arabidopsis (A. thaliana) cytosol (retrieved from Hirst et al, 2011), and human (H. sapiens) cytosol (retrieved from Uniprot). Domains of these proteins were extracted using Prosite and Pfam, then run against the chloroplast protein dataset to identify proteins putatively involved in vesicle transport inside chloroplasts. (PDF) [file pone.0104423.s006.pdf]

**Table S6.** CCV AP5 complex proteins from Arabidopsis (*A. thaliana*) cytosol (retrieved from Hirst et al, 2011), and human (*H. sapiens*) cytosol (retrieved from Uniprot). Domains of these proteins were extracted using Prosite and Pfam, then run against the chloroplast protein dataset to identify proteins putatively involved in vesicle transport inside chloroplasts.

| Organism, Accession No., Uniprot ID | Prosite profile/ pattern, Entry No. | Chloroplast proteins, Prosite Entry No. | Pfam profile/pattern, Entry No. | Chloroplast proteins, Pfam Entry No. |
|---|---|---|---|---|
| **ζ subunit** | | | | |
| *A. thaliana*, At3g15160, Q8H1F1 | n.d. | - | AP-5 complex subunit, vesicle trafficking: PF14764 | PF14764: n.d. |
| *H. sapiens*, NP_055670.1, O43299 | n.d. | - | AP-5 complex subunit, vesicle trafficking: PF14764 | PF14764: n.d. |
| **β5 subunit** | | | | |
| *A. thaliana*, At3g19870, F4JCE9 | n.d. | - | n.d | - |
| *H. sapiens*, NP_612377.3, Q2VPB7 | n.d. | - | n.d | - |
| **µ5 subunit** | | | | |
| *A. thaliana*, At2g20790, Q8WOZ6 | Mu homology domain (MHD) profile: PS51072<br><br>Clathrin adaptor complexes medium chain signature 2: PS00991<br><br>EF-hand calcium-binding domain PS00018 | PS51072: At5g57460<br><br>PS00991: n.d.<br><br>PS00018: At1g02150, At1g18890, At1g33790, At2g35800, At2g44990, At3g17470, At3g50770, At4g21940, At5g06043, At5g22800, At5g42380 | Adaptor complexes medium subunit family: PF00928 | PF00928: n.d. |
| *H. sapiens*, NP_060699.3, Q9HOR1 | Mu homology domain (MHD) profile: PS51072 | PS51072: At5g57460 | Adaptor complexes medium subunit family: PF00928 | PF00928: n.d. |
| **σ5 subunit** | | | | |
| *H. sapiens*, NP_060817.1, Q9NUS5 | n.d. | - | AP-5 complex subunit sigma-1: PF15001 | PF15001: n.d. |

n.d., not detected
