# Supplementary material for: Bioinformatic Indications That COPI- and Clathrin-Based Transport Systems Are Not Present in Chloroplasts: An Arabidopsis Model
Source: PLoS One. 2014 Aug 19;9(8):e104423. doi: 10.1371/journal.pone.0104423 (PMC4138088; doi:10.1371/journal.pone.0104423)
Supplement: Table S7 — B-COPI subcomplex proteins from Arabidopsis (A. thaliana) cytosol (retrieved from Bassham et al, 2008) and yeast (S. cerevisiae), mouse (M. musculus) and human (H. sapiens) cytosol (retrieved from Uniprot). Domains of these proteins were extracted using Prosite and Pfam, then run against the chloroplast protein dataset to identify proteins putatively involved in vesicle transport inside chloroplasts. (PDF) [file pone.0104423.s007.pdf]

Table S7. B-COPI subcomplex proteins from Arabidopsis (*A. thaliana*) cytosol (retrieved from Bassham et al, 2008) and yeast (*S. cerevisiae*), mouse (*M. musculus*) and human (*H. sapiens*) cytosol (retrieved from Uniprot). Domains of these proteins were extracted using Prosite and Pfam, then run against the chloroplast protein dataset to identify proteins putatively involved in vesicle transport inside chloroplasts.

**Organism, Accession No., Uniprot ID**	**Prosite profile/pattern, Entry No.**	**Chloroplast proteins, Prosite Entry No.**	**Pfam profile/pattern, Entry No.**	**Chloroplast proteins, Pfam Entry No.**
**α subunit**				
A. thaliana, At1g62020, Q94A40	Trp-Asp (WD) repeats profile: PS50082<br><br>Trp-Asp (WD) repeats circular profile: PS50294<br><br>Trp-Asp (WD) repeats signature: PS00678	PS50082: At1g18080, At2g33340, At4g22910, At5g13840, At1g24130, At4g02660, At5g58230, At1g15850<br><br>PS50294: At1g18080, At2g33340, At5g13840, At1g24130, At4g22910, At5g58230, At1g15850, At4g02660<br><br>PS00678: n.d.	WD domain, G-beta repeat: PF00400<br><br>Coatomer WD associated region: PF04053<br><br>Coatomer (COPI) alpha subunit C-terminus: PF06957	PF00400: At1g18080, At1g24130, At2g33340, At5g58230, At4g22910, At5g13840, At1g15850, At4g02660, At4g30840<br><br>PF04053: n.d.<br><br>PF06957: n.d.
A. thaliana, At2g21390, Q9SJT9	Trp-Asp (WD) repeats profile: PS50082<br><br>Trp-Asp (WD) repeats circular profile: PS50294<br><br>Trp-Asp (WD) repeats signature: PS00678	PS50082: At1g18080, At2g33340, At4g22910, At5g13840, At1g24130, At4g02660, At5g58230, At1g15850<br><br>PS50294: At1g18080, At2g33340, At5g13840, At1g24130, At4g22910, At5g58230, At1g15850, At4g02660<br><br>PS00678: n.d.	WD domain, G-beta repeat: PF00400<br><br>Coatomer WD associated region: PF04053<br><br>Coatomer (COPI) alpha subunit C-terminus: PF06957	PF00400: At1g18080, At1g24130, At2g33340, At5g58230, At4g22910, At5g13840, At1g15850, At4g02660, At4g30840<br><br>PF04053: n.d.<br><br>PF06957: n.d.
S. cerevisiae, COP1 YDL145C, P53622	Trp-Asp (WD) repeats profile: PS50082<br><br>Trp-Asp (WD) repeats circular profile: PS50294	PS50082: At1g18080, At2g33340, At4g22910, At5g13840, At1g24130, At4g02660,	WD domain, G-beta repeat: PF00400<br><br>Coatomer WD associated region: PF04053	PF00400: At1g18080, At1g24130, At2g33340, At5g58230, At4g22910, At5g13840, At1g15850, At4g02660, At4g30840<br><br>PF04053: n.d.

	Trp-Asp (WD) repeats signature: PS00678	At5g58230, At1g15850<br><br>PS50294: At1g18080, At2g33340 At5g13840, At1g24130, At4g22910, At5g58230, At1g15850, At4g02660<br><br>PS00678: n.d.	Coatomer (COPI) alpha subunit C-terminus: PF06957	PF06957: n.d.
**β' subunit**				
A. thaliana, At1g52360, Q9C827	Trp-Asp (WD) repeats profile: PS50082<br><br>Trp-Asp (WD) repeats circular profile: PS50294	PS50082: At1g18080, At2g33340, At5g13840, At1g24130, At4g22910, At5g58230, At1g15850, At4g02660<br><br>PS50294: At1g18080, At2g33340, At5g13840, At1g24130, At4g22910, At5g58230, At1g15850, At4g02660	WD domain, G-beta repeat: PF00400<br><br>Coatomer WD associated region: PF04053	PF00400: At1g18080, At1g24130, At2g33340, At5g58230, At4g22910, At5g13840, At1g15850, At4g02660, At4g30840<br><br>PF04053: n.d.
A. thaliana, At3g15980, Q8L828	Trp-Asp (WD) repeats profile: PS50082<br><br>Trp-Asp (WD) repeats circular profile: PS50294	PS50082: At1g18080, At2g33340, At5g13840, At1g24130, At4g22910, At5g58230, At1g15850, At4g02660<br><br>PS50294: At1g18080, At2g33340, At5g13840, At1g24130, At4g22910, At5g58230, At1g15850, At4g02660	WD domain, G-beta repeat: F00400<br><br>Coatomer WD associated region: PF04053	PF00400: At1g18080, At1g24130, At2g33340, At5g58230, At4g22910, At5g13840, At1g15850, At4g02660, At4g30840<br><br>PF04053: n.d.
A. thaliana, At1g79990, Q9CAA0	Trp-Asp (WD) repeats profile: PS50082	PS50082: At1g18080, At2g33340,	WD domain, G-beta repeat: F00400	PF00400: At1g18080, At1g24130, At2g33340, At5g58230, At4g22910,

	Trp-Asp (WD) repeats circular profile: PS50294	At5g13840, At1g24130, At4g22910, At5g58230, At1g15850 At4g02660<br><br>PS50294: At1g18080, At2g33340, At5g13840, At1g24130, At4g22910, At5g58230, At1g15850, At4g02660	Coatomer WD associated region: PF04053	At5g13840, At1g15850, At4g02660, At4g30840<br><br>PF04053: n.d.
H. sapiens, COPB2, P35606	Trp-Asp (WD) repeats profile: PS50082<br><br>Trp-Asp (WD) repeats circular profile: PS50294	PS50082: At1g18080, At2g33340, At5g13840, At1g24130, At4g22910, At5g58230, At1g15850, At4g02660<br><br>PS50294: At1g18080, At2g33340, At5g13840, At1g24130, At4g22910, At5g58230, At1g15850, At4g02660	WD domain, G-beta repeat: F00400<br><br>Coatomer WD associated region: PF04053	PF00400: At1g18080, At1g24130, At2g33340, At5g58230, At4g22910, At5g13840, At1g15850, At4g02660, At4g30840<br><br>PF04053: n.d.
**ε subunit**				
A. thaliana, At2g34840, O64748	Regulator of chromosome condensation (RCC1) signature 2: PS00626<br><br>Ribosomal protein S2 signature 1: PS00962	PS00626: n.d.<br><br>PS00962: n.d.	Coatomer epsilon subunit: PF04733	PF04733: n.d.
A. thaliana, At1g30630, Q9SA78	Ribosomal protein S2 signature 1: PS00962	PS00962: n.d.	Coatomer epsilon subunit: PF04733	PF04733: n.d.
H. sapiens, COPE, O14579	n.d.	-	Coatomer epsilon subunit: PF04733	PF04733: n.d.

n.d., not detected
