# Supplementary material for: Bioinformatic Indications That COPI- and Clathrin-Based Transport Systems Are Not Present in Chloroplasts: An Arabidopsis Model
Source: PLoS One. 2014 Aug 19;9(8):e104423. doi: 10.1371/journal.pone.0104423 (PMC4138088; doi:10.1371/journal.pone.0104423)
Supplement: Table S8 — F-COPI subcomplex proteins from Arabidopsis (A. thaliana) cytosol (retrieved from Bassham et al, 2008) and yeast (S. cerevisiae), mouse (M. musculus) and human (H. sapiens) cytosol (retrieved from Uniprot). Domains of these proteins were extracted using Prosite and Pfam, then run against the chloroplast protein dataset to identify proteins putatively involved in vesicle transport inside chloroplasts. (PDF) [file pone.0104423.s008.pdf]

Table S8. F-COPI subcomplex proteins from Arabidopsis (*A. thaliana*) cytosol (retrieved from Bassham et al, 2008) and yeast (*S. cerevisiae*), mouse (*M. musculus*) and human (*H. sapiens*) cytosol (retrieved from Uniprot). Domains of these proteins were extracted using Prosite and Pfam, then run against the chloroplast protein dataset to identify proteins putatively involved in vesicle transport inside chloroplasts.

**Organism, Accession No., Uniprot ID**	**Prosite profile/ pattern, Entry No.**	**Chloroplast proteins, Prosite Entry No.**	**Pfam profile/pattern, Entry No.**	**Chloroplast proteins, Pfam Entry No.**
**β subunit**				
A. thaliana, At4g31480, Q9SV21	EGF-like domain signature 1: PS00022	PS00022: n.d.	Adaptin N terminal region: PF01602<br><br>Coatomer beta C-terminal region: PF07718<br><br>Coatomer beta subunit appendage platform: PF14806	PF01602: At4g34450, At1g51350<br><br>PF07718: n.d.<br><br>PF14806: n.d.
A. thaliana, At4g31490, Q9SV20	EGF-like domain signature 1: PS00022	PS00022: n.d.	Adaptin N terminal region: PF01602<br><br>Coatomer beta C-terminal region: PF07718<br><br>Coatomer beta subunit appendage platform: PF14806	PF01602: At4g34450, At1g51350<br><br>PF07718: n.d.<br><br>PF14806: n.d.
S. cerevisiae, SEC26 YDR238C, P41810	n.d.	-	Adaptin N terminal region: PF01602<br><br>Coatomer beta C-terminal region: PF07718<br><br>Coatomer beta subunit appendage platform: PF14806	PF01602: At4g34450, At1g51350<br><br>PF07718: n.d.<br><br>PF14806: n.d.
**γ subunit**				
A. thaliana, At4g34450, Q0WW26	2-oxo acid dehydrogenases acyltransferase component lipoyl binding site: PS00189	PS00189: n.d.	Adaptin N terminal region: PF01602<br><br>Coatomer gamma subunit appendage platform subdomain: PF08752	PF01602: At4g34450, At1g51350<br><br>PF08752: At4g34450
S. cerevisiae, SEC21 YNL287W, P32074	n.d.	-	Adaptin N terminal region: PF01602	PF01602: At4g34450, At1g51350<br><br>PF08752: At4g34450

			Coatomer gamma subunit appendage platform subdomain: PF08752	
**δ subunit**				
A. thaliana, At5g05010, Q93Y22	Mu homology domain (MHD) profile: PS51072	PS51072: At5g57460	Adaptor complexes medium subunit family: PF00928	PF00928: n.d.
S. cerevisiae, RET2, P43621	Mu homology domain (MHD) profile: PS51072	PS51072: At5g57460	Clathrin adaptor complex small chain: PF01217<br><br>Adaptor complexes medium subunit family: PF00928	PF01217: n.d.<br><br>PF00928: n.d.
**ζ subunit**				
A. thaliana, At1g60970, Q940S5	n.d.	-	Clathrin adaptor complex small chain: PF01217	PF01217: n.d.
A. thaliana, At3g09800, Q84LG4	n.d.	-	Clathrin adaptor complex small chain: PF01217	PF01217: n.d.
A. thaliana, At1g08520, Q9SJE1	VWFA domain profile: PS50234	PS50234: At1g08520, At1g67120	Magnesium chelatase, subunit ChlI: PF01078<br><br>von Willebrand factor type A domain: PF13519	PF01078: At5g45930, At4g18480, At1g08520, At1g67120, At2g25140, At3g48870, At5g50920, At3g56690<br><br>PF13519: At1g08520
S. cerevisiae, RET3, P53600	n.d.	-	Clathrin adaptor complex small chain: PF01217	PF01217: n.d.

n.d., not detected
