# Supplementary material for: Bioinformatic Indications That COPI- and Clathrin-Based Transport Systems Are Not Present in Chloroplasts: An Arabidopsis Model
Source: PLoS One. 2014 Aug 19;9(8):e104423. doi: 10.1371/journal.pone.0104423 (PMC4138088; doi:10.1371/journal.pone.0104423)
Supplement: Table S9 — Coat GTPase proteins from Arabidopsis (A. thaliana) cytosol (retrieved from Bassham et al, 2008) and yeast (S. cerevisiae), mouse (M. musculus) and human (H. sapiens) cytosol (retrieved from Uniprot). Domains of these proteins were extracted using Prosite and Pfam, then run against the chloroplast protein dataset to identify proteins putatively involved in vesicle transport inside chloroplasts. (PDF) [file pone.0104423.s009.pdf]

Table S9. Coat GTPase proteins from Arabidopsis (*A. thaliana*) cytosol (retrieved from Bassham et al, 2008) and yeast (*S. cerevisiae*), mouse (*M. musculus*) and human (*H. sapiens*) cytosol (retrieved from Uniprot). Domains of these proteins were extracted using Prosite and Pfam, then run against the chloroplast protein dataset to identify proteins putatively involved in vesicle transport inside chloroplasts.

**Organism, Accession No., Uniprot ID**	**Prosite profile/ pattern, Entry No**	**Chloroplast proteins, Prosite Entry No.**	**Pfam profile/pattern, Entry No.**	**Chloroplast proteins, Pfam Entry No.**
**ArfA group**				
A. thaliana, At1g23490, Q9LQC8	small GTPase Arf family profile: PS51417	PS51417: At1g09180	ADP-ribosylation factor family: PF00025	PF00025: At1g09180, At1g05810, At4g35860, At5g57960
A. thaliana, At5g14670, Q9LYJ3	small GTPase Arf family profile: PS51417	PS51417: At1g09180	ADP-ribosylation factor family: PF00025	PF00025: At1g09180, At1g05810, At4g35860, At5g57960
A. thaliana, At2g47170, P36397	small GTPase Arf family profile: PS51417	PS51417: At1g09180	ADP-ribosylation factor family: PF00025	PF00025: At1g09180, At1g05810, At4g35860, At5g57960
A. thaliana, At1g70490, P0DH91	small GTPase Arf family profile: PS51417	PS51417: At1g09180	ADP-ribosylation factor family: PF00025	PF00025: At1g09180, At1g05810, At4g35860, At5g57960
A. thaliana, At3g62290, Q9M1P5	small GTPase Arf family profile: PS51417	PS51417: At1g09180	ADP-ribosylation factor family: PF00025	PF00025: At1g09180, At1g05810, At4g35860, At5g57960
A. thaliana, At1g10630, Q6ID97	small GTPase Arf family profile: PS51417	PS51417: At1g09180	ADP-ribosylation factor family: PF00025	PF00025: At1g09180, At1g05810, At4g35860, At5g57960
S. cerevisiae, ARF2, P19146	small GTPase Arf family profile: PS51417	PS51417: At1g09180	ADP-ribosylation factor family: PF00025	PF00025: At1g09180, At1g05810, At4g35860, At5g57960
**ArfB group**				
A. thaliana, At2g15310, Q9SHU5	small GTPase Arf family profile: PS51417	PS51417: At1g09180	ADP-ribosylation factor family: PF00025	PF00025: At1g09180, At1g05810, At4g35860, At5g57960
S. cerevisiae, ARF1, P11076	small GTPase Arf family profile: PS51417	PS51417: At1g09180	ADP-ribosylation factor family: PF00025	PF00025: At1g09180, At1g05810, At4g35860, At5g57960
**ArfD group**				
A. thaliana, At1g02440, F4HXI5	small GTPase Arf family profile: PS51417	PS51417: At1g09180	ADP-ribosylation factor family: PF00025	PF00025: At1g09180, At1g05810, At4g35860, At5g57960
A. thaliana, At1g02430, Q9FZ18	small GTPase Arf family profile: PS51417	PS51417: At1g09180	ADP-ribosylation factor family: PF00025	PF00025: At1g09180, At1g05810, At4g35860, At5g57960

S. cerevisiae, ARF1, P11076	small GTPase Arf family profile: PS51417	See above (PS51417: At1g09180)	ADP-ribosylation factor family: PF00025	PF00025: At1g09180, At1g05810, At4g35860, At5g57960
**ArfB2 group**				
A. thaliana, At5g17060, Q9LFJ7	small GTPase Arf family profile: PS51417	PS51417: At1g09180	ADP-ribosylation factor family: PF00025	PF00025: At1g09180, At1g05810, At4g35860, At5g57960
A. thaliana, At3g03120, Q9M9N1	small GTPase Arf family profile: PS51417	PS51417: At1g09180	ADP-ribosylation factor family: PF00025	PF00025: At1g09180, At1g05810, At4g35860, At5g57960
S. cerevisiae, ARF1, P11076	small GTPase Arf family profile: PS51417	PS51417: At1g09180	ADP-ribosylation factor family: PF00025	PF00025: At1g09180, At1g05810, At4g35860, At5g57960
