# Supplementary material for: Bioinformatic Indications That COPI- and Clathrin-Based Transport Systems Are Not Present in Chloroplasts: An Arabidopsis Model
Source: PLoS One. 2014 Aug 19;9(8):e104423. doi: 10.1371/journal.pone.0104423 (PMC4138088; doi:10.1371/journal.pone.0104423)
Supplement: Table S10 — CCV and COPI proteins from Arabidopsis (A. thaliana) cytosol (retrieved from Bassham et al, 2008) and yeast (S. cerevisiae), mouse (M. musculus) and human (H. sapiens) cytosol (retrieved from Uniprot). Domains of these proteins were extracted using Prosite and Pfam, run against the rice (subsp. japonica) protein dataset to identify proteins with the same domains, then those putatively involved in vesicle transport in chloroplasts were identified using Target P, and listed. (PDF) [file pone.0104423.s010.pdf]

Table S10. CCV and COPI proteins from Arabidopsis (*A. thaliana*) cytosol (retrieved from Bassham et al, 2008) and yeast (*S. cerevisiae*), mouse (*M. musculus*) and human (*H. sapiens*) cytosol (retrieved from Uniprot). Domains of these proteins were extracted using Prosite and Pfam, run against the rice (subsp. *japonica*) protein dataset to identify proteins with the same domains, then those putatively involved in vesicle transport in chloroplasts were identified using Target P, and listed.

Organism, Accession No., Uniprot ID	Prosite profile/pattern, Entry No.	Chloroplast proteins, (reliability class), Prosite Entry No.,	Pfam profile/pattern, Entry No.	Chloroplast proteins, (reliability class), Pfam Entry No.
**CLATHRIN COATED VESICLE COMPONENTS**				
**Clathrin heavy chain**				
A. thaliana, At3g11130, Q0WNJ6	Clathrin heavy-chain (CHCR) repeat profile: PS50236<br><br>Orn/DAP/Arg decarboxylases family 2 pyridoxal-P attachment site: PS00878	PS50236 and PS00878: n.d.	Clathrin propeller repeat: PF01394<br><br>Clathrin, heavy-chain linker: PF09268<br><br>Clathrin-H-link: PF13838<br><br>Region in Clathrin and VPS: PF00637	PF01394, PF09268, PF13838 and PF00637: LOC_Os11g01380 (5), LOC_Os12g01390 (5)
A thaliana, At3g08530, Q0WLB5	Clathrin heavy-chain (CHCR) repeat profile: PS50236<br><br>Orn/DAP/Arg decarboxylases family 2 pyridoxal-P attachment site: PS00878	PS50236 and PS00878: n.d.	Clathrin propeller repeat: PF01394<br><br>Clathrin, heavy-chain linker: PF09268<br><br>Clathrin-H-link: PF13838<br><br>Region in Clathrin and VPS: PF00637	PF01394, PF09268, PF13838 and PF00637: LOC_Os11g01380 (5), LOC_Os12g01390 (5)
S. cerevisiae, CHC1 YGL206C, P22137	Clathrin heavy-chain (CHCR) repeat profile: PS50236	PS50236: LOC_Os11g01380 (5), LOC_Os12g01390 (5)	Clathrin, heavy-chain linker: PF09268<br><br>Clathrin-H-link: PF13838<br><br>Region in Clathrin and VPS: PF00637	PF09268, PF13838 and PF00637: LOC_Os11g01380 (5), LOC_Os12g01390 (5)
**Clathrin light chain**				
A. thaliana, At2g40060, O04209	n.d.	-	Clathrin light chain: PF01086	PF01086: detected but none in chloroplasts
A. thaliana, At3g51890, F4J5M9	n.d.	-	Clathrin light chain: PF01086	PF01086: detected but none in chloroplasts
S. cerevisiae, CLC1 YGR167W, P17891	Clathrin light chain signature 2: PS00581	PS00581: n.d.	Clathrin light chain: PF01086	PF01086: detected but none in chloroplasts

AP1 SUBUNITS				
**AP1 γ subunit**				
A. thaliana, At1g60070, Q9ZUI6	Gamma-adaptin ear (GAE) domain profile: PS50180	PS50180: n.d.	Adaptin N terminal region: PF01602<br><br>Adaptin C-terminal domain: PF02883	PF01602 and PF02883: detected but none in chloroplasts
A. thaliana, At1g23900, Q84K16	Gamma-adaptin ear (GAE) domain profile: PS50180	PS50180: n.d.	Adaptin N terminal region: PF01602<br><br>Adaptin C-terminal domain: PF02883	PF01602 and PF02883: detected but none in chloroplasts
S. cerevisiae, APL4 YPR029C, Q12028	Peroxidases proximal heme-ligand signature: PS00435	PS00435: LOC_Os04g35520 (3), LOC_Os02g34810 (1)	Adaptin N terminal region: PF01602<br><br>Adaptin C-terminal domain: PF02883	PF01602 and PF02883: detected but none in chloroplasts
**AP1 β1 subunit***				
S. cerevisiae, APL2 YKL135C, P36000	n.d.	-	Adaptin N terminal region: PF01602	PF01602: LOC_Os01g43630 (5), LOC_Os01g32880 (5), LOC_Os11g41990 (5)
**AP1 β1/β2 subunit**				
A. thaliana, At4g11380, Q9SUS3	n.d.	-	Adaptin N terminal region: PF01602<br><br>Adaptin C-terminal domain: PF02883<br><br>Beta2-adaptin appendage, C-terminal sub-domain: PF09066	PF01602, PF02883 and PF09066: detected but none in chloroplasts
A. thaliana, At4g23460, O81742	n.d.	-	Adaptin N terminal region: PF01602<br><br>Adaptin C-terminal domain: PF02883<br><br>Beta2-adaptin appendage, C-terminal sub-domain: PF09066	PF01602, PF02883 and PF09066: detected but none in chloroplasts
**AP1 µ1 subunit**				
A. thaliana, At1g60780, O22715	Mu homology domain (MHD) profile: PS51072<br><br>Clathrin adaptor complexes	PS51072, PS00990 and PS00991: n.d.	Clathrin adaptor complex small chain: PF01217<br><br>Adaptor complexes	PF01217 and PF00928: detected but none in chloroplasts

	medium chain signature 1: PS00990<br><br>Clathrin adaptor complexes medium chain signature 2: PS00991		medium subunit family: PF00928	
A. thaliana, At1g10730, Q9SAC9	Mu homology domain (MHD) profile: PS51072<br><br>Clathrin adaptor complexes medium chain signature 2: PS00991	PS51072 and PS00991: n.d.	Clathrin adaptor complex small chain: PF01217<br><br>Adaptor complexes medium subunit family: PF00928	PF01217 and PF00928: detected but none in chloroplasts
S. cerevisiae, APM1 YPL259C, Q00776	Mu homology domain (MHD) profile: PS51072<br><br>Clathrin adaptor complexes medium chain signature 1: PS00990<br><br>Clathrin adaptor complexes medium chain signature 2: PS00991	PS51072, PS00990, PS00991: n.d.	Clathrin adaptor complex small chain: PF01217<br><br>Adaptor complexes medium subunit family: PF00928	PF01217 and PF00928: detected but none in chloroplasts
**AP1 σ1 subunit**				
A. thaliana, At2g17380, Q8LEZ8	Clathrin adaptor complexes small chain signature: PS00989	PS00989: n.d.	Clathrin adaptor complex small chain: PF01217	PF01217: detected but none in chloroplasts
A. thaliana, At4g35410, O23685	Clathrin adaptor complexes small chain signature: PS00989	PS00989: n.d.	Clathrin adaptor complex small chain: PF01217	PF01217: detected but none in chloroplasts
M. musculus, Aps1s1, P61967	Clathrin adaptor complexes small chain signature: PS00989	PS00989: n.d.	Clathrin adaptor complex small chain: PF01217	PF01217: detected but none in chloroplasts
		**AP2 SUBUNITS**		
**AP2 α subunit**				
A. thaliana, At5g22770, Q8LPL6	n.d.	-	Adaptin N terminal region: PF01602<br><br>Adaptin C-terminal domain: PF02883<br><br>Alpha adaptin AP2, C-terminal domain: PF02296	PF01602, PF02883 and PF02296: detected but none in chloroplasts
A. thaliana,	n.d.	-	Adaptin N	PF01602, PF02883 and

At5g22780, Q8LPK4			terminal region: PF01602<br><br>Adaptin C-terminal domain: PF02883<br><br>Alpha adaptin AP2, C-terminal domain: PF02296	PF02296: detected but none in chloroplasts
S. cerevisiae, APL3 YBL037W, P38065	n.d.	-	Adaptin N terminal region: PF01602<br><br>Adaptin C-terminal domain: PF02883	PF01602 and PF02883: detected but none in chloroplasts
**AP2 β2 subunit***				
S. cerevisiae, APL1 YJR005W, P27351	Carbamoyl-phosphate synthase subdomain signature 2: PS00867	PS00867: LOC_Os05g22940 (2), LOC_Os01g38970 (3)	Adaptin N terminal region: PF01602	PF01602: LOC_Os01g43630 (5), LOC_Os01g32880 (5), LOC_Os11g41990 (5)
**AP2 β1/β2 subunit**	See above	See above	See above	See above
**AP2 μ2 subunit**				
A. thaliana, At5g46630, B9DI54	Mu homology domain (MHD) profile: PS51072	PS51072: detected but none in chloroplasts	Adaptor complexes medium subunit family: PF00928	PF00928: LOC_Os12g34370
S. cerevisiae, APM4 YOL062C, Q99186	Mu homology domain (MHD) profile: PS51072	PS51072: detected but none in chloroplasts	Adaptor complexes medium subunit family: PF00928	PF00928: LOC_Os12g34370
**AP2 σ2 subunit**				
A. thaliana, At1g47830, Q84WL9	n.d.	-	Clathrin adaptor complex small chain: PF01217	PF01217: detected but none in chloroplasts
S. cerevisiae, APS2 YJR058C, Q00381	Clathrin adaptor complexes small chain signature: PS00989	n.d.	Clathrin adaptor complex small chain: PF01217	PF01217: detected but none in chloroplasts
**AP3 SUBUNITS**				
**AP3 δ subunit**				
A. thaliana, At1g48760, Q9C744	n.d.	-	Adaptin N terminal region: PF01602	PF01602: LOC_Os01g43630 (5), LOC_Os01g32880 (5), LOC_Os11g41990 (5)
S. cerevisiae, APL5 YPL195W, Q08951	n.d.	-	Adaptin N terminal region: PF01602	PF01602: LOC_Os01g43630 (5), LOC_Os01g32880 (5), LOC_Os11g41990 (5)
**AP3 β3 subunit**				
A. thaliana, At3g55480, Q9M2T1	n.d.	-	Adaptin N terminal region: PF01602	PF01602: LOC_Os01g43630 (5), LOC_Os01g32880 (5), LOC_Os11g41990 (5)
S. cerevisiae, APL6	n.d.	-	Adaptin N terminal region:	PF01602: LOC_Os01g43630 (5),

YGR261C, P46682			PF01602	LOC_Os01g32880 (5), LOC_Os11g41990 (5)
**AP3 μ3 subunit**				
A. thaliana, At1g56590, Q8LPJ0	Mu homology domain (MHD) profile: PS51072<br><br>Clathrin adaptor complexes medium chain signature 1: PS00990	PS51072 and PS00990: n.d.	Adaptor complexes medium subunit family: PF00928	PF00928: LOC_Os12g34370
S. cerevisiae, APM3 YBR288C, P38153	Mu homology domain (MHD) profile: PS51072<br><br>Clathrin adaptor complexes medium chain signature 1: PS00990<br><br>Clathrin adaptor complexes medium chain signature 2: PS00991	PS51072, PS00990 and PS00991: n.d.	Adaptor complexes medium subunit family: PF00928	PF00928: LOC_Os12g34370
**AP3 σ3 subunit**				
A. thaliana, At3g50860, Q8VZ37	Clathrin adaptor complexes small chain signature: PS00989	PS00989: n.d.	Clathrin adaptor complex small chain: PF01217	PF01217: detected but none in chloroplasts
S. cerevisiae, APS3 YJL024C, P47064	Clathrin adaptor complexes small chain signature: PS00989	PS00989: n.d.	Clathrin adaptor complex small chain: PF01217	PF01217: detected but none in chloroplasts
**AP4 SUBUNITS**				
**AP4 ε subunit**				
A. thaliana, At1g31730, Q8L7A9	n.d.	-	Adaptin N terminal region: PF01602	PF01602: LOC_Os01g43630 (5), LOC_Os01g32880 (5), LOC_Os11g41990 (5)
H. sapiens, AP4E1, Q9UPM8	n.d.	-	Adaptin N terminal region: PF01602<br><br>Adaptin AP4 complex epsilon appendage platform: PF14807	PF01602 and PF14807: n.d.
**AP4 β4 subunit**				
A. thaliana, At5g11490, Q9LDK9	n.d.	-	Adaptin N terminal region: PF01602<br><br>Beta2-adaptin appendage, C-terminal sub-domain: PF09066	PF01602 and PF09066: LOC_Os01g43630 (5)

M. musculus, Ap4b1, Q9WV76	n.d.	-	Adaptin N terminal region: PF01602<br><br>Beta2-adaptin appendage, C-terminal sub-domain: PF09066	PF01602 and PF09066: LOC_Os01g43630 (5)
**AP4 µ4 subunit**				
A. thaliana, At4g24550, Q9SB50	Mu homology domain (MHD) profile: PS51072	PS51072: detected but none in chloroplasts	Clathrin adaptor complex small chain: PF01217<br><br>Adaptor complexes medium subunit family: PF00928	PF01217 and PF00928: detected but none in chloroplasts
H. sapiens, AP4M1, O00189	Mu homology domain (MHD) profile: PS51072<br><br>Clathrin adaptor complexes medium chain signature 2: PS00991	PS51072 and PS00991: n.d.	Clathrin adaptor complex small chain: PF01217<br><br>Adaptor complexes medium subunit family: PF00928	PF01217 and PF00928: detected but none in chloroplasts
**AP4 σ4 subunit**				
A. thaliana, At4g24550, Q9SB50	Mu homology domain (MHD) profile: PS51072	PS51072: detected but none in chloroplasts	Clathrin adaptor complex small chain: PF01217<br><br>Adaptor complexes medium subunit family: PF00928	PF01217: detected but none in chloroplasts<br><br>PF00928: detected but none in chloroplasts
H. sapiens, AP4S1, Q9Y587	n.d.	-	Clathrin adaptor complex small chain: PF01217	PF01217: detected but none in chloroplasts
**AP5 SUBUNITS**				
**AP5 ζ subunit**				
A. thaliana, At3g15160, Q8H1F1	n.d.	-	AP-5 complex subunit, vesicle trafficking: PF14764	PF14764: n.d.
H. sapiens, NP_055670.1, O43299	n.d.	-	AP-5 complex subunit, vesicle trafficking: PF14764	PF14764: n.d.
**AP5 β5 subunit**				
A. thaliana, At3g19870, F4JCE9	n.d.	-	n.d	-
H. sapiens, NP_612377.3, Q2VPB7	n.d.	-	n.d	-
**AP5 µ5 subunit**				
A. thaliana, At2g20790, Q8WOZ6	Mu homology domain (MHD) profile: PS51072	PS51072, PS00991 and PS00018: n.d.	Adaptor complexes medium subunit family: PF00928	PF00928: LOC_Os12g34370

	Clathrin adaptor complexes medium chain signature 2: PS00991<br><br>EF-hand calcium-binding domain PS00018			
H. sapiens, NP_060699.3, Q9HOR1	Mu homology domain (MHD) profile: PS51072	PS51072: detected but none in chloroplasts	Adaptor complexes medium subunit family: PF00928	PF00928: LOC_Os12g34370
**AP5 σ5 subunit**				
H. sapiens, NP_060817.1, Q9NUS5	n.d.	-	AP-5 complex subunit sigma-1: PF15001	PF15001: n.d.
**COPI COMPONENTS**				
**B-COPI SUBUNITS**				
**B-COPI α subunit**				
A. thaliana, At1g62020, Q94A40	Trp-Asp (WD) repeats profile: PS50082<br><br>Trp-Asp (WD) repeats circular profile: PS50294<br><br>Trp-Asp (WD) repeats signature: PS00678	PS50082, PS50294 and PS00678: LOC_Os03g46650 (3)	WD domain, G-beta repeat: PF00400<br><br>Coatomer WD associated region: PF04053<br><br>Coatomer (COPI) alpha subunit C-terminus: PF06957	PF00400, PF04053 and PF06957: n.d.
A. thaliana, At2g21390, Q9SJT9	Trp-Asp (WD) repeats profile: PS50082<br><br>Trp-Asp (WD) repeats circular profile: PS50294<br><br>Trp-Asp (WD) repeats signature: PS00678	PS50082, PS50294 and PS00678: LOC_Os03g46650 (3)	WD domain, G-beta repeat: PF00400<br><br>Coatomer WD associated region: PF04053<br><br>Coatomer (COPI) alpha subunit C-terminus: PF06957	PF00400, PF04053 and PF06957: n.d.
S. cerevisiae, COP1 YDL145C, P53622	Trp-Asp (WD) repeats profile: PS50082<br><br>Trp-Asp (WD) repeats circular profile: PS50294<br><br>Trp-Asp (WD) repeats signature: PS00678	PS50082, PS50294 and PS00678: LOC_Os03g46650 (3)	WD domain, G-beta repeat: PF00400<br><br>Coatomer WD associated region: PF04053<br><br>Coatomer (COPI) alpha subunit C-terminus: PF06957	PF00400, PF04053 and PF06957: n.d.
**B-COPI β' subunit**				
A. thaliana,	Trp-Asp (WD)	PS50082 and PS50294:	WD domain, G-	PF00400 and PF04053:

At1g52360, Q9C827	repeats profile: PS50082<br><br>Trp-Asp (WD) repeats circular profile: PS50294	LOC_Os03g46650 (3)<br>LOC_Os12g01922 (4)	beta repeat: PF00400<br><br>Coatomer WD associated region: PF04053	n.d.
A. thaliana, At3g15980, Q8L828	Trp-Asp (WD) repeats profile: PS50082<br><br>Trp-Asp (WD) repeats circular profile: PS50294	PS50082 and PS50294:<br>LOC_Os03g46650 (3)<br>LOC_Os12g01922 (4)	WD domain, G-beta repeat: F00400<br><br>Coatomer WD associated region: PF04053	PF00400 and PF04053: n.d.
A. thaliana, At1g79990, Q9CAA0	Trp-Asp (WD) repeats profile: PS50082<br><br>Trp-Asp (WD) repeats circular profile: PS50294	PS50082 and PS50294:<br>LOC_Os03g46650 (3)<br>LOC_Os12g01922 (4)	WD domain, G-beta repeat: F00400<br><br>Coatomer WD associated region: PF04053	PF00400 and PF04053: n.d.
H. sapiens, COPB2, P35606	Trp-Asp (WD) repeats profile: PS50082<br><br>Trp-Asp (WD) repeats circular profile: PS50294	PS50082 and PS50294:<br>LOC_Os03g46650 (3)<br>LOC_Os12g01922 (4)	WD domain, G-beta repeat: F00400<br><br>Coatomer WD associated region: PF04053	PF00400 and PF04053: n.d.
**B-COPI ε subunit**				
A. thaliana, At2g34840, O64748	Regulator of chromosome condensation (RCC1) signature 2: PS00626<br><br>Ribosomal protein S2 signature 1: PS00962	PS00626 and PS00962: n.d.	Coatomer epsilon subunit: PF04733	PF04733: LOC_Os07g14530 (5)
A. thaliana, At1g30630, Q9SA78	Ribosomal protein S2 signature 1: PS00962	PS00962: detected but none in chloroplasts	Coatomer epsilon subunit: PF04733	PF04733: LOC_Os07g14530 (5)
H. sapiens, COPE, O14579	n.d.	-	Coatomer epsilon subunit: PF04733	PF04733: LOC_Os07g14530 (5)
		**F-COPI SUBUNITS**		
**F-COPI β subunit**				
A. thaliana, At4g31480, Q9SV21	EGF-like domain signature 1: PS00022	PS00022: detected but none in chloroplasts	Adaptin N terminal region: PF01602<br><br>Coatomer beta C-terminal region: PF07718<br><br>Coatomer beta subunit appendage platform: PF14806	PF01602, PF07718 and PF14806: n.d.
A. thaliana, At4g31490, Q9SV20	EGF-like domain signature 1: PS00022	PS00022: detected but none in chloroplasts	Adaptin N terminal region: PF01602	PF01602, PF07718 and PF14806: n.d.

			Coatomer beta C-terminal region: PF07718<br><br>Coatomer beta subunit appendage platform: PF14806	
S. cerevisiae, SEC26 YDR238C, P41810	n.d.	-	Adaptin N terminal region: PF01602<br><br>Coatomer beta C-terminal region: PF07718<br><br>Coatomer beta subunit appendage platform: PF14806	PF01602, PF07718 and PF14806: n.d.
**F-COPI γ subunit**				
A. thaliana, At4g34450, Q0WW26	2-oxo acid dehydrogenases acyltransferase component lipoyl binding site: PS00189	PS00189: detected but none in chloroplasts	Adaptin N terminal region: PF01602<br><br>Coatomer gamma subunit appendage platform subdomain: PF08752	PF01602 and PF08752: n.d.
S. cerevisiae, SEC21 YNL287W, P32074	n.d.	-	Adaptin N terminal region: PF01602<br><br>Coatomer gamma subunit appendage platform subdomain: PF08752	PF01602 and PF08752: n.d.
**F-COPI δ subunit**				
A. thaliana, At5g05010, Q93Y22	Mu homology domain (MHD) profile: PS51072	PS51072: detected but none in chloroplasts	Adaptor complexes medium subunit family: PF00928	PF00928: LOC_Os12g34370
S. cerevisiae, RET2, P43621	Mu homology domain (MHD) profile: PS51072	PS51072: detected but none in chloroplasts	Clathrin adaptor complex small chain: PF01217<br><br>Adaptor complexes medium subunit family: PF00928	PF01217 and PF00928: detected but none in chloroplasts
**F-COPI ζ subunit**				
A. thaliana, At1g60970,	n.d.	-	Clathrin adaptor complex small	PF01217: detected but none in chloroplasts

Q940S5			chain: PF01217	
A. thaliana, At3g09800, Q84LG4	n.d.	-	Clathrin adaptor complex small chain: PF01217	PF01217: detected but none in chloroplasts
A. thaliana, At1g08520, Q9SJE1	VWFA domain profile: PS50234	PS50234: LOC_Os03g59640 (5)	Magnesium chelatase, subunit ChlI: PF01078<br><br>von Willebrand factor type A domain: PF13519	PF01078 and PF13519: n.d.
S. cerevisiae, RET3, P53600	n.d.	-	Clathrin adaptor complex small chain: PF01217	PF01217: detected but none in chloroplasts
**COAT GTPase PROTEINS**				
**ArfA group**				
A. thaliana, At1g23490, Q9LQC8	small GTPase Arf family profile: PS51417	PS51417: detected but none in chloroplasts	ADP-ribosylation factor family: PF00025	PF00025: LOC_Os03g59590 (5)
A. thaliana, At5g14670, Q9LYJ3	small GTPase Arf family profile: PS51417	PS51417: detected but none in chloroplasts	ADP-ribosylation factor family: PF00025	PF00025: LOC_Os03g59590 (5)
A. thaliana, At2g47170, P36397	small GTPase Arf family profile: PS51417	PS51417: detected but none in chloroplasts	ADP-ribosylation factor family: PF00025	PF00025: LOC_Os03g59590 (5)
A. thaliana, At1g70490, P0DH91	small GTPase Arf family profile: PS51417	PS51417: detected but none in chloroplasts	ADP-ribosylation factor family: PF00025	PF00025: LOC_Os03g59590 (5)
A. thaliana, At3g62290, Q9M1P5	small GTPase Arf family profile: PS51417	PS51417: detected but none in chloroplasts	ADP-ribosylation factor family: PF00025	PF00025: LOC_Os03g59590 (5)
A. thaliana, At1g10630, Q6ID97	small GTPase Arf family profile: PS51417	PS51417: detected but none in chloroplasts	ADP-ribosylation factor family: PF00025	PF00025: LOC_Os03g59590 (5)
S. cerevisiae, ARF2, P19146	small GTPase Arf family profile: PS51417	PS51417: detected but none in chloroplasts	ADP-ribosylation factor family: PF00025	PF00025: LOC_Os03g59590 (5)
**ArfB group**				
A. thaliana, At2g15310, Q9SHU5	small GTPase Arf family profile: PS51417	PS51417: detected but none in chloroplasts	ADP-ribosylation factor family: PF00025	PF00025: LOC_Os03g59590 (5)
S. cerevisiae, ARF1, P11076	small GTPase Arf family profile: PS51417	PS51417: detected but none in chloroplasts	ADP-ribosylation factor family: PF00025	PF00025: LOC_Os03g59590 (5)
**ArfD group**				
A. thaliana, At1g02440, F4HXI5	small GTPase Arf family profile: PS51417	PS51417: detected but none in chloroplasts	ADP-ribosylation factor family: PF00025	PF00025: LOC_Os03g59590 (5)
A. thaliana, At1g02430, Q9FZ18	small GTPase Arf family profile: PS51417	PS51417: detected but none in chloroplasts	ADP-ribosylation factor family: PF00025	PF00025: LOC_Os03g59590 (5)
S. cerevisiae, ARF1, P11076	small GTPase Arf family profile: PS51417	PS51417: detected but none in chloroplasts	ADP-ribosylation factor family: PF00025	PF00025: LOC_Os03g59590 (5)
**ArfB2 group**				
A. thaliana, At5g17060,	small GTPase Arf family profile:	PS51417: detected but none in chloroplasts	ADP-ribosylation factor family:	PF00025: LOC_Os03g59590 (5)

Q9LFJ7	PS51417		PF00025	
A. thaliana, At3g03120, Q9M9N1	small GTPase Arf family profile: PS51417	PS51417: detected but none in chloroplasts	ADP-ribosylation factor family: PF00025	PF00025: LOC_Os03g59590 (5)
S. cerevisiae, ARF1, P11076	small GTPase Arf family profile: PS51417	PS51417: detected but none in chloroplasts	See above (ADP-ribosylation factor family: PF00025)	PF00025: LOC_Os03g59590 (5)

n.d., not detected
